# Prefrontal connectomics: from anatomy to human imaging

**DOI:** 10.1038/s41386-021-01156-6

**Published:** 2021-09-28

**Authors:** Suzanne N. Haber, Hesheng Liu, Jakob Seidlitz, Ed Bullmore

**Affiliations:** 1 Department of Pharmacology and Physiology, University of Rochester School of Medicine & Dentistry, Rochester, NY 14642 USA; 2 Department of Psychiatry, McLean Hospital, Harvard Medical School, Belmont, MA 02478 USA; 3 Department of Neuroscience, Medical University of South Carolina, Charleston, SC USA; 4 Department of Radiology, Massachusetts General Hospital, Harvard Medical School, Boston, MA USA; 5 Department of Psychiatry, University of Pennsylvania, Philadelphia, USA; 6 Department of Psychiatry, University of Cambridge, Herchel Smith Building for Brain and Mind Sciences, Cambridge Biomedical Campus, Cambridge, CB2 0SZ UK

**Keywords:** Neuroscience, Nervous system

## Abstract

The fundamental importance of prefrontal cortical connectivity to information processing and, therefore, disorders of cognition, emotion, and behavior has been recognized for decades. Anatomic tracing studies in animals have formed the basis for delineating the direct monosynaptic connectivity, from cells of origin, through axon trajectories, to synaptic terminals. Advances in neuroimaging combined with network science have taken the lead in developing complex wiring diagrams or connectomes of the human brain. A key question is how well these magnetic resonance imaging (MRI)-derived networks and hubs reflect the anatomic “hard wiring” first proposed to underlie the distribution of information for large-scale network interactions. In this review, we address this challenge by focusing on what is known about monosynaptic prefrontal cortical connections in non-human primates and how this compares to MRI-derived measurements of network organization in humans. First, we outline the anatomic cortical connections and pathways for each prefrontal cortex (PFC) region. We then review the available MRI-based techniques for indirectly measuring structural and functional connectivity, and introduce graph theoretical methods for analysis of hubs, modules, and topologically integrative features of the connectome. Finally, we bring these two approaches together, using specific examples, to demonstrate how monosynaptic connections, demonstrated by tract-tracing studies, can directly inform understanding of the composition of PFC nodes and hubs, and the edges or pathways that connect PFC to cortical and subcortical areas.

## Introduction

The modern era of neuroanatomy began with the development of cellular and axonal markers at the turn of the twentieth century. Nissl and Golgi stains could, for the first time, allow scientists to visualize the morphology of cells and permit the classification of cell types. Myelin degenerative stains made possible the visualization of myelinated axons and the connections between brain regions. Based on these and other stains developed at the end of the nineteenth century, two anatomic subfields for understanding brain organization emerged and blossomed during the early twentieth century: cytoarchitectonics (led by Brodmann and others), which segmented the brain based on cortical layer cell morphology; and myeloarchitectonics (led primarily by the Vogts), which classified cortical areas based on myelin distribution and fiber orientation through cortical layers [1–3]. However, the modern ideas about the functional specification of brain regions, and the importance of communication between regions, predate these technical developments and maps. Indeed, they can be traced back to the anatomist Franz J Gall (1758–1828), who not only assigned function to regions of gray matter but also recognized the functional significance of white matter (WM) connectivity between regions [4]. Carl Wernicke (1848–1905), the father of disconnection theory, promoted the idea that connectivity was central to function, and that functions were not localized within specific brain regions (with the exception of primary sensory and motor functions) [5]. Geschwind modified this view by suggesting that higher cortical function involves a combination of functional localization and connectivity between specialized cortical areas, leading to the idea that the brain was comprised of complex anatomic networks supporting cognitive and emotional processes [6]. The term “hubs” was first used in human network neuroscience to describe the critical role of transmodal cortical areas as “anatomical and computational epicenters for large-scale neurocognitive networks” [7] (Box 1).

More recently, advances in neuroimaging have been combined with network science in an effort to quantify the complex wiring diagram of whole nervous systems, or connectomes. Whole-brain functional magnetic resonance imaging (fMRI) networks have been subdivided into functionally specialized resting-state networks, including the default mode network (DMN) [8]. Graph theoretical analysis has offered a more quantitative approach to the concept of hubs and it has been recognized that highly connected hubs are central to integrative processing across brain networks [9], and also create vulnerabilities for dysfunction [10]. Thus, computational analyses of connectivity, based on imaging data, have formalized the definition of hubs and led to the identification of several brain regions that likely integrate diverse information [11]. Together, these emerging ideas of brain organization have laid the foundation for new research approaches for delineating and probing brain networks, which may be disrupted in psychiatric disorders [12].

However, a key question is how well these MRI-derived networks and hubs reflect the anatomic “hard wiring” first proposed to underlie the distribution of information for large-scale network interactions. Although much of the work on brain networks originated in the anatomic literature, MRI studies have now taken the lead in this endeavor. Indeed, there has been an explosion of interest in linking psychiatric illnesses to circuit dysfunction in one or more of the resting-state networks measured by functional resting-state functional MRI (rs-fMRI), or the metrics of anatomical connectivity measured by diffusion-weighted MRI (dMRI) or other structural MRI modalities. Overall, such studies raise interesting and important questions about the dynamic and structural changes or abnormalities within and across networks in vivo [13, 14]. Coupled with computational approaches, these methods provide powerful tools to uncover the nodes, hubs, and edges (connections) that constitute information processing networks. Indeed, several rs-fMRI networks have been identified that contain hubs within the prefrontal cortex (PFC), including but not limited to the ventral (VAN) and dorsal (DAN) attention networks, the salience network (SN), an executive network (frontoparietal), and perhaps the most widely studied networks of all—the DMN [8, 15–21].

However, all available methods of MRI of the living brain are indirect methods of measuring connectivity. Thus, the question remains how precisely brain activity or connections inferred from MRI data are linked to monosynaptic connections. In contrast, non-human primate (NHP) anatomic tracing studies allow direct visualization of monosynaptic connectivity, from cells of origin, through axon trajectories, to synaptic terminals. This fine resolution of anatomical hard wiring provides the ability to identify the composition of nodes and hubs embedded within relatively large cortical regions defined as nodes at the relatively coarse resolution provided by neuroimaging. This potentially allows for a better definition of the critical integrative regions or network hubs and a more precise evaluation of the fiber bundles likely to connect the nodes or hubs. Linking this anatomic “gold standard” to the connectivity profiles derived from the indirect, imaging methods in humans will be a key next step to better understand the locations of network nodes and connections that are related to disease and therefore could be targeted for therapeutic interventions.

A key challenge is to translate what we know about monosynaptic connectivity from the anatomical studies in animals to inform and constrain the interpretation of functional and structural connectivity metrics derived from multimodal MRI measurements. In this review, we address this challenge by focusing on what is known about monosynaptic prefrontal cortical connections in NHPs and how this compares to MRI-derived measurements of network organization in humans. We use the NHP as our animal model of prefrontal cortical connectivity since it is most closely homologous to the human PFC (see Preuss and Wise in this volume). First, we set the stage by outlining the direct approach to identifying anatomic connections by tract tracing and review the major monosynaptic connections and pathways for each PFC region. We then review the available MRI-based techniques for indirectly measuring structural and functional connectivity and introduce graph theoretical methods for the analysis of hubs, modules, and topologically integrative features of the connectome. Finally, we bring these two approaches together, demonstrating how monosynaptic connections demonstrated by tract-tracing studies can directly inform understanding of the nodes and hubs comprised by the PFC, and the edges or pathways that connect PFC nodes with other cortical and subcortical areas. In this section, we focus on the two most widely used MRI methods for anatomical and functional connectivity mapping, dMRI, and rs-fMRI, respectively. We highlight two examples of anatomically defined long-distance pathways or edges that connect the PFC to subcortical targets: the anterior limb of the internal capsule (ALIC) and the dopamine–PFC pathway. Although these pathways do not link corticocortical connections between hubs, they exemplify an approach that allows more precise translation between anatomic and imaging methods and is generalizable to corticocortical tracts. Moreover, the ALIC is an excellent example of a long-distance fiber bundle that carries all PFC fibers, and it is the principal target for neurosurgical lesions and deep brain stimulation (DBS) therapeutic approaches for obsessive-compulsive disorder and depression (see Rasmussen and Goodman in this volume) [22–28]. The midbrain dopamine system is arguably the most studied system in psychiatry and is increasingly a focus of imaging studies. Finally, we use the DMN, with a focus on its hub in the medial PFC, to determine the anatomical composition of this hub and to test the consistency of both dMRI- and rs-fMRI-based metrics of connectivity with anatomic connections defined by tract tracing. As noted, the DMN is one of the most widely studied human brain networks in both normal and clinically abnormal states [8, 29–34].

Box 1 Terminology

- *Circuits and networks*: are generally used to denote a set of paths between two or more nodes or brain areas. Anatomic circuits traditionally refer to a sequence of directed monosynaptic connections. In neuroimaging, the term is typically used to identify undirected connections between regions that are statistically coupled or correlated in some way. These connections do not necessarily reflect monosynaptic anatomical connections.
- *Hub*: was first used as a term to describe the role of highly connected brain regions in mediating information flow across the whole-brain network, inferred from anatomic studies of directed monosynaptic connectivity [7]. The term is now widely used in the imaging literature to describe nodes with a high degree of connectivity, but not necessarily mediated by monosynaptic connections.
- *Pathways and edges*: the anatomically defined connections that interconnect cortical and subcortical areas or nodes are commonly also referred to as pathways that travel in specific WM tracts or bundles. Edges are more broadly defined as the links between nodes in a graph theoretical or graphical model of a network. An edge may be equivalent to a pathway if the graph is based on anatomic tract-tracing data, but, if the graph is based on MRI data, an edge may also represent a statistical measure of connectivity between nodes without implying that this represents an underlying axonal pathway.
- *Distances and paths*: distance is a spatial or geometric measure (in units of mm or cm), whereas a path is topologically defined. In tract-tracing data, the distance traversed by curvilinear axonal tracts can be directly measured, whereas in MRI data the anatomical distance between regions is often approximated by the Euclidean (straight line) distance between them. The distance of axonal tracts is also often used as a proxy measure of wiring cost. Long-distance connections, e.g., between occipital and prefrontal cortices or between bilaterally homologous regions of the temporal cortex, can be regarded as more costly than the more frequent, short-distance connections between neighboring and ipsilateral regions of PFC or other cortical lobes. Paths, in contrast, are measured in terms of the integer number of edges that connect two nodes, or the number of synapses that mediate information flow between them. Thus, if two widely separated areas of the cortex are monosynaptically connected, the distance between them will be long but the path length will be short.

## Tract-tracing methods for identifying the synaptic-based organization of the frontal cortex

Prior to the early 1950s, the only available methods for tracing connections, in addition to dissection, were histological stains that identified degenerating myelin sheaths following well-placed lesions. This method did not identify unmyelinated or thinly myelinated axons, or terminals. In the 1950s, a reduced silver method was developed that did not depend on myelin, but was instead sensitive to the axons themselves. However, this was shortly replaced by even more sensitive techniques that depended on active neuronal transport, including tracers that were preferentially transported anterogradely (e.g., tritiated amino acids) or retrogradely (e.g., horseradish peroxidase conjugated to wheat germ agglutin. Soon other molecules were identified with intrinsic fluorescence, whose sensitivity could be further increased with immunohistochemical processing. These tracers have in turn now largely been replaced by viral tracers, optogenetic methods, etc., especially for rodent work. Collectively, these methods clearly allow the direct visualization of labeled cells, axons, their pathways through the WM, and terminal fields.

However, there are important limitations to these methods. First, and perhaps most important, they can only be used in animals. Thus, direct visualization of connections from one brain region to another is not possible in humans, for which only indirect methods (e.g., imaging) are available. Tracers suffer from a variety of other problems. Conventional tracers can be taken up by fibers of passage and the exact area of axonal uptake at the injection site can be difficult to determine. In addition, inconsistency in uptake and transport can result in variability. Nonetheless, the large, cumulative literature that contains hundreds of tracer injections in different PFC regions using these conventional tracers clearly demonstrates the organization of PFC connections and the replicability of these connections across laboratories. Due to the high costs and variability of viral tracers and optogenetic methods in NHPs, large studies replicating the anatomic literature are neither practical nor needed.

## Frontocortical connectivity and pathways

The PFC is a large, complex, and heterogeneous area that can be broadly subdivided into regions that include the dorsolateral PFC (dlPFC), ventrolateral PFC (vlPFC), rostral parts of the orbitofrontal cortex (OFC), and frontal pole or area 10 (FP). In addition, the caudal OFC and anterior cingulate cortex (ACC) are often also included in the PFC, despite the fact that, based on the cytoarchitectonic criteria (the presence of a clear granular layer), they may not be technically classified as PFC. For a more complete discussion of PFC classification, see Preuss and Wise in this volume. As these areas are implicated in many psychiatric illnesses, we include them in this review. In addition to these classically recognized anatomic areas, a ventral and medial region of PFC (the ventromedial PFC or vmPFC) has more recently been identified primarily based on imaging studies that demonstrated the region’s activation during both positive and negative decision-making tasks and implicated it in several diseases [35–41]. However, the area is not well-defined anatomically and different studies include various anatomic areas within the vmPFC. For example, some researchers include only ventral areas 10 and 32 [42], others include rostromedial orbital area 11, while others include more lateral and caudal OFC areas [43]. Due to this lack of consistency across studies, in this review, we focus on the anatomic areas within the vmPFC, rather than a broader vmPFC region.

**Fig. 1:**
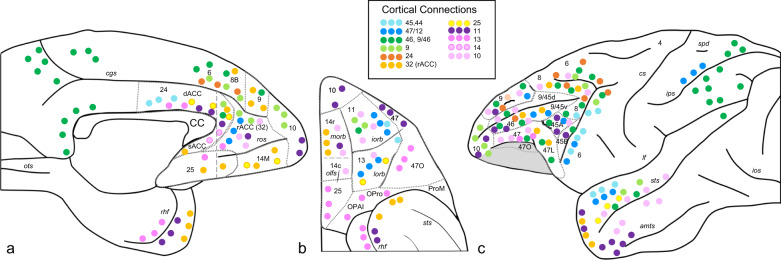
A general overview of the denser connections to prefrontal areas. **a** Sagittal view, **b** orbital view, and **c** lateral view. For a more complete connectional description for each area, see the references listed in section “Frontocortical connectivity and pathways” of the review. Each dot represents connections from the corresponding cortical area indicated by its color. Blue indicates inputs from vlPFC (light blue = areas 45 and 44, dark blue = area 47); green indicates inputs form dlPFC (light green = area 9; dark green = areas 46 and 9/46); red/orange indicates inputs from ACC (red = area 24, orange = area 32, unfilled dots = area 25; purple/pink indicates inputs from OFC (dark purple = area 11, dark pink = area 14, light pink = area 14); pale pink indicates frontal pole (area 10). amts anterior middle temporal sulcus, CC corpus callosum, cgs cingulate sulcus, cs central sulcus, dACC dorsal anterior cingulate cortex, ios inferior occipital sulcus, ips intraparietal sulcus, lf lateral fissure, lorb lateral orbital sulcus, morb medial orbital sulcus, olfs olfactory sulcus, OPAI orbital periallocortex, Opro orbital proisocortex, ots occipitotemporal sulcus, ProM area ProM (promotor), rACC rostral anterior cingulate cortex, rhf rhinal fissure, ros rostral sulcus, sACC subgenual anterior cingulate cortex, spd superior postcentral dimple, sts superior temporal sulcus.

Each PFC region is further architectonically divided into specific cortical areas. Based on the widely used Brodman’s areas, these include dlPFC, areas 9, 46, and 9/46; vlPFC, areas 44, 45, and 47/12; ACC, areas 24, 25, and 32; and OFC, areas 11, 13, and 14. The connections of these areas and the pathways they use to reach their targets are well documented [24, 44–53]. Overall, each cortical region is highly connected to adjacent areas (referred to as short-distance connections), but axons also travel long distances to reach other specific cortical regions (long-distance connections). Thus, e.g., axons from area 10 project to adjacent areas 9, 11, and 32 and also make long-distance connections to the superior temporal cortex. Likewise, area 9/46 has short-distance connections to areas 9, 46, vlPFC, and premotor cortex, as well as long-distance connections to the parietal cortex, etc. (Fig. 1).

**Fig. 2:**
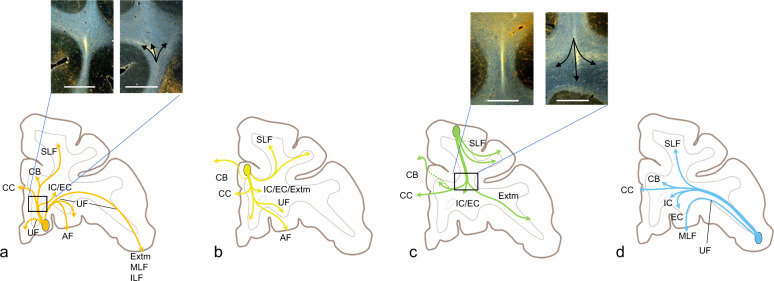
Schematic demonstrating axons entering the white matter from ventral, medial, dorsal, and lateral PFC regions and branching to enter different pathways. **a** Fibers exiting the OFC. Box indicates the inset of photomicrographs of the stalk and branching axons. **b** Fibers exiting the anterior cingulate cortex. **c** Fibers exiting the dorsolateral PFC. Box indicates the inset of photomicrographs of stalk and branching axons. **d** Fibers exiting the ventrolateral prefrontal cortex. AF amygdalofugal pathway, CB cingulum bundle, CC corpus callosum, EC external capsule, Extm extreme capsule, IC internal capsule, IL inferior longitudinal fasciculus, MLF medial longitudinal fasciculus, SLF superior longitudinal fasciculus, UF uncinate fasciculus.

Although there is a large anatomic literature describing cortical and subcortical connections of the PFC, fewer studies have focused on the organization of the axons as they travel through the WM. However, this understanding is now critical as more emphasis is placed on WM as an important component of circuit dysfunction in psychiatric illnesses and as a therapeutic target. Axons exit each cortical area and are organized in a similar patterned arrangement. One set of fibers forms a dense, centrally located stalk as it enters the WM [24, 44, 54]. The stalk carries the capsular fibers (internal (IC), external, and extreme (Extm) capsules), corpus callosum (CC), cingulate bundle (CB), and Muratoff bundle (corticostriatal fibers) (Fig. 2). Short- and long-distance ipsilateral corticocortical fibers travel adjacent to the stalk as they enter the major WM pathways [24, 44, 54]. Long-distance connections from dorsal and more caudal PFC areas travel primarily through the superior longitudinal fasciculus (SLF I, II, and III), Extm, the CC, and CB. Those from ventral regions travel primarily through the Extm and uncinate fasciculus (UF), CC, and CB. Subcortical connections to the thalamus and brainstem travel in the IC and through the Extm and Muratoff bundle to reach the striatum [24, 44, 47, 48, 55–58]. Below, we briefly outline the main connections and pathways from each region, with some additional examples of the trajectories of some key fiber pathways.

### Orbitofrontal cortex

OFC has unique access to both primary and highly processed sensory information. These connections, coupled with those to the amygdala, cingulate, and perirhinal cortex, explain many of the functional properties of the OFC. Overall, it is considered to play an essential role in learning complex stimulus–outcome relationships and signaling outcome expectations. While other brain regions are also involved in these relationships, the OFC is particularly critical for value-based decision making, as this requires updating value-based changes over time [12, 59–62]. The OFC is a primary node for the limbic network with direct connections to both cortical and subcortical regions that modulate motivation and emotions. For a more complete discussion of OFC function, see Izquierdo and Rudebeck in this volume.

The OFC occupies the ventral surface of the frontal lobe and is bounded medially by area 10 and the subgenual ACC (sACC) (rostrally and caudally, respectively) and laterally by the vlPFC (see Fig. 1b). The OFC can be generally parceled into somewhat different regions based on a rostrocaudal axis and a mediolateral axis of connections and functions [45, 63–65]. The most medial part of the orbital surface (gyrus rectus) includes areas 14 and ventral 10. Areas 11 (rostrally) and 13 (caudally) are bounded laterally by the lateral OFC sulci. Lateral to the lateral OFC sulcus is the orbital portion of the area of the vlPFC (area 47/12). The caudal OFC region (area 13) and adjacent agranular areas (see Preuss and Wise in this volume) receive direct inputs from the primary olfactory and gustatory cortex, as well as higher-order somatosensory, auditory, and visual cortical areas. Taken together, the caudal OFC is considered important for integrating input from multisensory regions [63–65]. In addition to these sensory inputs, perirhinal cortex, an area important for object recognition, also projects primarily to the caudal OFC [66]. In contrast to the caudal areas, the rostral OFC (area 11), which is the granular cortex, receives not only highly processed sensory information but is also connected to cognitive areas of the frontal lobe, including the FP and rostral areas 9 and 24, and lateral areas 45 and 46. Interestingly, the parahippocampal gyrus also projects primarily to the rostral OFC [52, 64].

Consistent with these connectional differences between caudal and rostral OFC, there is an apparent gradient between primary reward representations in more caudal OFC/insular cortex and representations of secondary rewards, such as money, in more rostral OFC regions [67]. Such dissociations may rely on the differential inputs from early versus higher sensory representations to caudal versus rostral OFC, respectively, or they may reflect the preponderance of amygdala connections to caudal OFC compared to the rostral OFC connections to other regions, such as dlPFC and FP, which are more important for executive functions. There is also a mediolateral gradient of connections. Medial regions of OFC are tightly linked to medial PFC, including the cingulate cortex, medial area 10, and medial area 9. Lateral OFC areas are heavily connected to the vlPFC and area 46. These connectional differences, as with the rostrocaudal gradient, are likely related to the different behavioral effects of lesions that involve more medial versus lateral OFC areas [62]. As with all PFC connections described below, this is an overview of general OFC connections. Indeed, within any given area, there are connections with multiple regions, including some outside the expected areas. For example, while the entorhinal cortex and the subiculum project primarily to the caudal OFC, some terminal patches are also found in rostral areas.

Pathways from the ventral surface of the frontal cortex enter the UF immediately adjacent to their cortical region, some of them forming the stalk, which cuts through the UF to enter the capsules and striatum. Others travel medially through the UF to enter the CB, and the CC (Fig. 2a). Axons enter the IC and striatum from a ventral position, passing through the Extm that surrounds the striatum, terminating in the ventral striatum and taking up a ventral position in the IC. A medial bundle travels within the UF to reach medial orbital regions, the sACC, and the CC and CB. Laterally, fibers continue in the UF to innervate lateral orbital regions, the temporal pole, entorhinal cortex, and subiculum. As orbital fibers enter the temporal lobe, some branch laterally to enter the middle longitudinal fasciculus terminating in the superior temporal gyrus [24, 44, 47, 55, 68].

### Anterior cingulate cortex

In contrast to the OFC, the ACC has a relative absence of sensory connections, but is tightly connected to emotional, cognitive, and motor control areas. The ACC lies on the medial surface, extending from the level of the premotor cortex, curving rostrally with the genu of the CC and then caudally, ventral to the callosum. It is composed of multiple subregions that support its wide range of functions, including the use of value-related information to help regulate flexibility, adaptation, and top-down control [69–73]. For a more complete discussion of ACC function, see Monosov and Rushworth in this volume. This functionally heterogeneous region is further subdivided into the sACC (areas 25, 32), the rostral ACC (rACC, areas 32, 24), and dorsal ACC (dACC, area 24) (see Fig. 1a).

The sACC is an important mediator of emotion, motivation, and determining value [71, 74–76] and is connected to the motivation network including the medial and caudal OFC, hippocampus, hypothalamus, and amygdala. The rACC is located rostral to the genu of the CC and is tightly linked with the sACC, dACC, OFC (area 14), medial area 9, the dlPFC and vlPFC, and the rostral temporal cortex. More dorsocaudal regions of the rACC have some links to rostral motor control areas, including the frontal eye fields (FEFs) and the prepresupplementary motor area [52, 77]. Thus, the rACC sits at the connectional intersection of the motivation and action control networks, an important position in the transition from valuation through choice to action, particularly in situations of uncertainty [69, 78–82]. The rACC is considered a hub and one of the main anchors within the DMN [8, 83, 84]. Located caudal to the genu, the dACC is more tightly connected with the action network consisting of motor control areas, including FEF and premotor areas [52, 77], and is associated with motor planning and action execution [85, 86]. This region also has strong connections to the insula and is considered to be part of the SN defined by rs-fMRI [16] (see also Menon and D’Esposito in this volume). Interestingly, amygdala projections continue to terminate in patches throughout the dACC, including some in the caudal parts [66]. Importantly, the connections of these divisions through the ACC are a continuum and there are no specific borders between the ACC subdivisions [77].

Cingulate fibers forming the stalk cut through the CB to reach frontal WM before splitting into pathways that are directed towards the CC, the capsules, and striatum (Fig. 2b). Axons within the IC occupy a position dorsal to those from the OFC, preserving a dorsal–ventral topography within the capsule [24]. These fibers are also positioned in the medial part of the IC. ACC fibers traveling to PFC regions do not form a single bundle, but continue to cross through the CB as a continuous stream of fibers and course along its edge, arching around the gyrus to reach dorsal medial and lateral frontal areas. Thus, fibers emerging from a given ACC region traveling to ipsilateral and contralateral cortical areas, the striatum, thalamus, and brainstem, do not remain within the CB. However, those dACC axons that do join the dorsal CB travel rostrally and caudally for significant distances to terminate throughout the cingulate cortex, including the posterior cingulate cortex. Fibers also travel ventrally within the subgenual CB to reach the sACC. Some of these axons also join the UF to terminate in the OFC. Others join the amygdalofugal pathway and medial forebrain bundle (MFB) to terminate in the amygdala and hypothalamus [44, 47, 56].

### Frontal pole (area 10)

This region is large in human, but relatively small in NHPs, and perhaps the least studied of PFC areas. Functionally, it is considered to be involved in higher cognitive function, complex reasoning, and abstract rule representation [87–89]. Based on NHP studies, the region is considered as the apex of cognitive function in the PFC hierarchy [90], while other studies suggest that its central role in mediating the balance between exploitation and exploration [89]. The frontopolar area is strongly connected to rostral frontal regions (areas 9, 46, 32, 24, 11, and 14). Less densely connected regions are located caudally in the orbital area 13, vlPFC areas 47/12 and 45, premotor area 6, and area 8. Unlike adjacent areas 9 and 46, area 10 has dense connections to the temporal lobe, including auditory, visual, and multisensory association regions [44, 47, 91]. Fibers from the frontal pole take both a dorsal route through the SLF and CB, and a ventral route through the Extm [47]. These pathways are topographically organized, with fibers from dorsal regions taking similar routes as fibers from area 9, and ventral regions using similar pathways as the OFC (unpublished observations).

### Dorsolateral PFC

The dlPFC is further divided into areas 9, 46, and 9/46 (see Fig. 1c). The dlPFC is involved in executive function and specifically in working memory [92–94]. Although other brain regions are also active during the delay phase of working memory tasks (including the vlPFC), activity in the dlPFC, especially more caudal PFC regions surrounding the principal sulcus in areas 46 and 9/46, is particularly linked with the action associated with working memory task [95–97]. For a more complete discussion of the role of dlPFC in cognitive control see Friedman and Robbins in this volume. Areas 46 and 9/46 have tight links to adjacent frontal areas, including the FEFs, premotor cortex, and vlPFC (areas 44, 45 and 47/12). There are additional, albeit fewer, connections with orbital regions, particularly area 11. Long-distance connections of dlPFC are primarily with the cingulate cortex, including posterior cingulate cortex, retrosplenial cortex, and with both inferior and superior parietal lobules. Connections with the parietal cortex form the basis for the frontoparietal network involved in cognitive control [98, 99]. Finally, there are some, albeit less-dense connections with the superior temporal sulcus, rostral temporal, and paralimbic regions. Area 9 has similar connections, except that it conspicuously lacks connections with the parietal cortex. Thus, in contrast to areas 46 and 9/46, rostral 9 is primarily connected to adjacent PFC regions, including area 10 and ACC, although there are also some connections to OFC and temporal cortex [100–104].

Fibers from the dlPFC form the stalk curve around the dorsal aspect of the striatum as they enter the IC, where they are positioned dorsal to those from the vlPFC and lateral to fibers from the medial PFC (Fig. 2c). A second group of fibers leaves the stalk and travel medially to join the CB and CC and a third group enters the Muratoff bundle. Short association fibers travel within the subcortical WM to innervate other dlPFC regions, area 10, area 8, and premotor cortex. Long association fibers enter the SLF (I, II, and III), Extm and UF. Cortical fibers traveling caudally enter the SLF (I and II). Those within the SLF I terminate in the superior parietal cortex and secondary somatosensory area II. Others travel more ventrally, through the SLF II, to reach the caudal inferior parietal lobule and those within the SLF III terminate in the frontoparietal opercular cortex. Fibers traveling to the temporal lobe travel through the Extm, to join the middle longitudinal fasciculus (Fig. 2c) [44, 47, 48, 101].

### Ventrolateral PFC

The vlPFC has been implicated in behavioral flexibility, memory retrieval, reversal learning, and language [105–111]. For a complete discussion of the vlPFC function, see Monosov and Rushworth in this volume. This multifunctional area benefits from its position in the frontal cortex, surrounded by (and connected to) areas involved in sensory, cognitive, emotional, and motor processing. The vlPFC is cytoarchitecturally and functionally divided into three areas, 47/12, 45, and 44 (Fig. 1c). Area 47/12 (pars orbitalis of the inferior frontal gyrus, IFG) is the most rostral and ventral region. Area 47/12 is considered a component of the ventral attention rs-fMRI network [112] and its anatomic connections place it in a key position between sensory, motor, and limbic systems. This large region has dense anatomic connections to multiple adjacent regions of the PFC, including the OFC medially (areas 11 and 13) and dorsally (areas 44, 45, 46, and 9/46), specific ACC regions, lateral area 9, and ventral area 6. Temporal lobe connections include the parahippocampal gyrus, perirhinal cortex, TE, TL, TA, TPO, and amygdala [24, 46, 48, 50, 66, 113–116]. Finally, area 47 has limited but functionally important connections with specific parietal regions, e.g., the intraparietal sulcus [46, 49, 101]. Areas 45 and 44 are caudal and dorsal to area 47, corresponding to the pars triangularis and pars opercularis of the IFG, respectively. These areas both participate in language functions and are more tightly linked to motor control regions (areas 8 and 6) compared to area 47 [50, 113, 117]. Although area 45 is also connected to the OFC and ACC, area 44 is not. Indeed, area 44 is most closely connected to motor control regions, and functionally important for motor inhibitory control [108, 118, 119].

Axons from caudal vlPFC areas split off from the stalk and curve around the dorsolateral aspect of the striatum to enter the IC where they are positioned between OFC (ventral) and dlPFC (dorsal) fibers and lateral to ACC axons. Other fibers continue medially to enter the exterme capsule, Extm, and striatum (Fig. 2d). Axons that enter the Extm travel dorsally to the dlPFC. Other fibers travel ventrally in the Extm merging with the UF to terminate in the OFC. Finally, some axons continue into the temporal cortex, merging with the middle longitudinal fasciculus (Fig. 2d) [44, 48].

### Fiber pathways carrying long-distance PFC connections

**Fig. 3:**
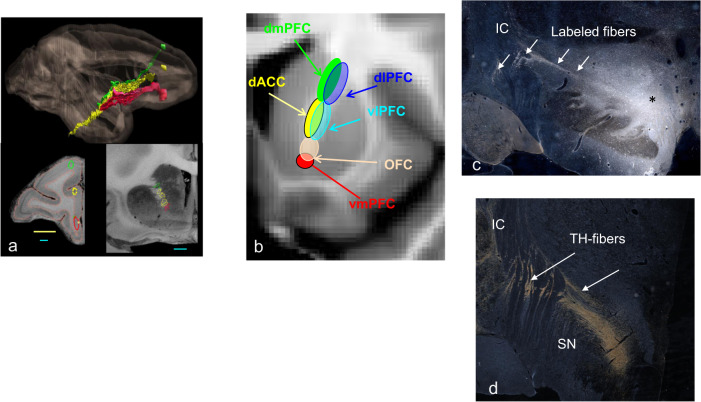
Pathways through the anterior limb of the internal capsule (a, b) and medial forebrain bundle (c, d). **a** Sagittal and coronal views of the dorsoventral positions of fibers from the medial wall (green = area 9m; yellow = area 24; red = area 14m) traveling through the ALIC; lower left image represents each injection site placement. **b** Segmentation of the ALIC. Coronal view: red = vmPFC; pink = OFC; yellow = dACC; teal = vlPFC; green = dmPFC; blue = dlPFC. **c** Tritiated amino acid injection into the ventral tegmental area with labeled fibers streaming laterally, crossing through the internal capsule. **d** Tyrosine hydroxylase-positive staining, illustrating the trajectory of dopamine axon similar to the (**c**). IC internal capsule, SN substantia nigra.

While each cortical area uses a different combination of fiber bundles to reach their targets, all PFC areas send fibers through the ALIC, and all receive ascending dopamine projections through the MFB. We highlight these two subcortical pathways as illustrative examples of how anatomy can inform interpretation of connectivity results from imaging. Moreover, these two bundles have particular significance in circuit dysfunction theories for psychiatry. The ALIC contains all the ascending and descending PFC fibers and has been a target for both neurosurgical lesions and DBS for the treatment of OCD and depression. The separation of ALIC fibers from the stalk as described above (see photomicrographs in Fig. 2) creates a problem for diffusion MRI streamlines to follow accurately. This will be discussed in greater detail in section “Connectomics: topological properties of brain networks modeled as graphs” below. The organization of the fibers within the ALIC is based on how axons from different cortical regions enter and are positioned within the capsule. vmPFC and OFC fibers enter the capsule ventrally and occupy the most ventral portion of the capsule; those from dorsal and lateral cortical regions (vlPFC, dlPFC, dACC, and dorsomedial PFC) enter the capsule dorsally and laterally and move ventrally. There is a dorsoventral topography, with fibers from dorsal regions positioned dorsal to those from ventral regions (Fig. 3a). In addition, fibers from medial areas are positioned medial to those from lateral areas. This organization results in the ability to segment the ALIC into five general regions based on PFC input (Fig. 3b) [55, 120]. As fibers descend in the capsule, those terminating in the thalamus are positioned medial to those that continue to the brainstem and spinal cord [55].

The MFB contains the ascending dopaminergic fibers that terminate throughout the frontal cortex, albeit with different distribution patterns [121, 122]. This bundle courses rostrally through the ventral forebrain, before arching dorsally around the CC to enter the frontal cortex [123–125]. In addition, fibers leave the ventral tegmental area and cross the IC to enter the striatum directly (Fig. 3c, d). As we will see below, this crossing becomes a challenge for tractography analysis of diffusion MRI data to accurately track the ventral tegmental dopamine fibers to the PFC [28], resulting in a false-positive streamline within the capsule. This false streamline has been erroneously named as part of the MFB [126, 127].

### Summary

All regions of the PFC, as defined above, are associated with different, albeit overlapping functions that are reflective of their complex interconnections. These include both short-distance connections surrounding each cortical area and long-distance connections, both within the frontal cortex and the temporal and parietal lobes. In addition to the corticocortical projections, axons from each area terminate in overlapping striatal and thalamic regions. Some PFC areas, notably the OFC, ACC, and vlPFC, are also tightly connected to the amygdala. Each cortical region is large and contains subregions with different functional and connectivity profiles.

The combination of MRI and statistical methods for the analysis of patterns of functional and anatomical connectivity across the whole brain has substantially advanced our understanding of global brain network organization. rs-fMRI data can be reliably decomposed into functionally specialized networks, many of which include distinct PFC areas as hubs for distributing information within or between networks [128]. The rACC is part of the DMN; dACC is part of the SN; the vlPFC is part of the ventral attention network; and the dlPFC is part of the executive, cognitive control, or multitask network [16, 19, 20, 29, 98] (see also Menon and D’Esposito in this volume). The DMN has been of particular focus in the detailed investigation since its discovery as a system of consistently task-deactivated regions in multiple fMRI activation studies [8]. Recent data have indicated that the DMN can be further divided into individually variable subnetworks in single-subject fMRI scans. Importantly, anatomically homologous regions can be identified as part of a putative DMN by fMRI studies in anesthetized rodents and NHPs. Tract-tracing studies in the marmoset have also been used to show that retrograde tracer injection into frontopolar regions (areas 9, 10, and 11) substantially recapitulates the connectivity of the DMN [83], indicating that these PFC areas of the marmoset brain are an input hub, receiving short-distance projections from other areas of PFC, as well as long-distance projections from the ACC, the posterior cingulate cortex, and the parietal cortex. These studies remind us that detailed reconciliation of tract-tracing and MRI data will ultimately require a fine-grained approach to areal parcellation, analysis of connections or edges as well as areas or nodes, and likely increasing emphasis on individual differences in the pattern of anatomical and functional connectivity characteristic of late-developing PFC areas.

We now summarize the MRI methods commonly used to assess anatomical and functional connectivity (section “Frontocortical connectivity and pathways”) and then introduce some graph theoretical methods for network analysis with a particular focus on how we can quantify hubs of high connectivity and define the organization of pathways or edges (section “MRI methods for indirectly measuring anatomical and functional connectivity”).

## MRI methods for indirectly measuring anatomical and functional connectivity

**Fig. 4:**
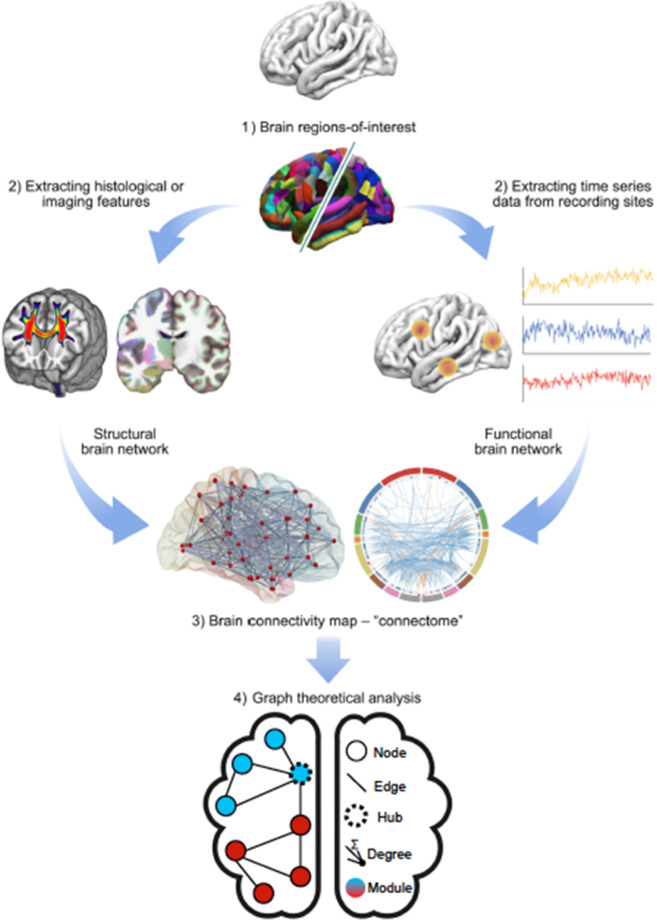
Human brain network analysis: high-level schematic. Brain regions or nodes are defined based on anatomical, functional, or multimodal parcellations, thus subdividing the whole brain into *p* regional nodes (1). Connectivity between nodes can be estimated in many different ways, to define the weight of an edge or connection between each possible pair of nodes. Anatomical connectivity (2, left) can be estimated by dMRI tractography, structural covariance, or morphometric similarity; functional connectivity (2, right) can be estimated by the correlation between nodal mean time series. The resulting (*p* × *p*) matrix is the connectome, which can be represented in diverse formats, including (left) an anatomical rendering, where the nodes are located at the centroid of each region and a line is drawn between nodes if their pairwise connectivity exceeds an arbitrary threshold; or (right) a ring diagram, where the regional nodes are arranged around the perimeter of the circle (color-coded according to anatomical criteria or modular affiliation) and edges traverse the interior of the circle denoting suprathreshold connectivity. Finally, the complex topological properties of these networks, including hubs and modules, can be estimated using tools from graph theory, here illustrated for the simplest class of binary undirected graphs (from [236]).

Methods for analyzing human brain networks in vivo have transformed the field, providing opportunities to characterize not only normal connections but also pathologies or changes associated with psychiatric disorders (Fig. 4). However, in contrast to anatomic tracing studies, dMRI and fMRI are indirect measures of network connectivity—neither the axons nor cells are labeled. Moreover, these methods do not indicate the directionality of connections.

### Diffusion-weighted MRI (dMRI)

Tractography, based on the diffusivity of water molecules constrained by tissue microstructure, reflects axonal orientation that is captured in terms of directionally polarized or anisotropic diffusion of water molecules by dMRI signals. Tractography algorithms use the local orientation information in each voxel to estimate the axonal tract trajectories from one voxel to the next, sometimes over long anatomical distances. Thus, tractography does not reconstruct axons but demonstrates the so-called “streamlines”, i.e., paths of least hindrance to water diffusion, which are assumed to run parallel with axonal tracts, and as such it is prone to error [129–133]. Tractography typically summarizes the local orientational information in the data in terms of a distribution function, with a small number of peaks indicative of dominant axonal orientations. But determining the axonal trajectories that give rise to these peaks is ambiguous because there are multiple configurations of axon populations that can give rise to the same peak distributions. As a result, a tractography algorithm working out the next step in the streamline will have to make an arbitrary decision on which peak to follow with more dominant directions emphasized, and smaller bundles (with different orientations) de-emphasized or lost. Simplistic rules on the geometry of bundles (e.g., to minimize the bending angle or the path length between regions) are typically used to make these decisions. These rules are unlikely to be true at every decision point in the voxel-by-voxel reconstruction of streamlines, which leads to errors in tractography such as false-positive connections [129, 134], or biases in the distributions of cortical terminations, i.e., gyral bias [132]. NHP tracing data can be used to elucidate the extent to which such rules are appropriate [24].

### Structural covariance network analysis and morphometric similarity

Anatomical connectivity has also been inferred from gray matter MRI measurements by two classes of methods: structural covariance network analysis and morphometric similarity analysis. Structural covariance is typically measured by the interregional correlation of a macrostructural scalar, e.g., cortical thickness or volume, estimated on the basis of MRI data from hundreds of people. High positive structural covariance indicates that two cortical regions tend to vary in size together across a group of brains. It is difficult to know how to interpret this metric in terms of the axo-synaptic connectivity of an individual brain. There is some support for the neurodevelopmental model [135, 136] that structural covariance reflects coupled growth of brain regions benefitting from the sustained mutually trophic effects of reciprocal axo-synaptic connectivity [137].

The morphometric similarity is typically measured by the interregional correlation of a macro- and/or microstructural vector, e.g., a cortical depth profile of multiple T1 or magnetization transfer (MT) measurements spanning the 2–3 mm distance between pial surface and WM boundary [138, 139], estimated on the basis of MRI data from a single individual. A high correlation between regional MRI feature vectors indicates that they are microstructurally similar in some way, e.g., two regions may share a similar depth profile of a cortical myelination marker like MT [139].

The first tier of biological interpretation is that such morphometrically similar regions are architectonically congruous, i.e., they have similar lamination and/or myelination of cortical tissue. This interpretation is supported by data showing that morphometric similarity (positive correlation measured using a regional pair of 10-feature vectors of macro- and microstructural MRI parameters in each individual scan) was generally greater between regions belonging to the same von Economo class of cortex, whereas dissimilarity (negative interregional correlation of feature vectors) was more likely between regions in distinct cytoarchitectonic areas [140].

The second tier of biological interpretation is that cyto- or myelo-architectonally congruent cortical areas are more likely to be reciprocally interconnected by axo-synaptic projections than dissimilar regions. This relationship is substantiated by prior histological data from primate cortical anatomy [141]. Thus, morphometric similarity measured by MRI can be conceived as a proxy marker of axo-synaptic connectivity between architectonically similar regions. This two-step interpretation of morphometric similarity as an indirect marker of anatomical connectivity has been experimentally tested, and to some extent validated, by comparison of morphometric similarity measured by MRI and axo-synaptic connectivity measured by tract tracing in the primate cortex [142].

### Resting-state functional MRI

**Fig. 5:**
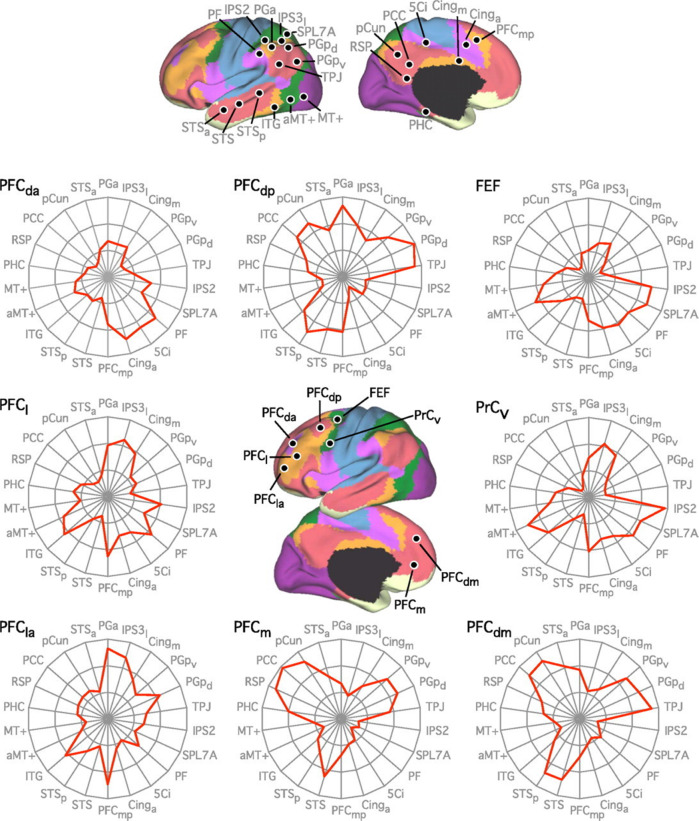
Functional connectivity profiles of human prefrontal cortical seed regions. Eight seed regions of lateral and medial PFC (shown in the central panel) were used to estimate the functional connectivity—or fMRI time-series correlations—between the seed and a range of other cortical regions. The eight radar plots show the strength of connectivity between each of the seeds (located at the center of the circles) and 22 other regions (labeled on the perimeter of each circle). The red line shows the strength of functional connectivity in the range −0.4 to 0.5, against the background of three concentric circles representing connectivity of −0.1 (the inner circle), +0.2 (the middle circle), or +0.5 (the outer circle or perimeter) (from [20]).

In addition to these three MRI-based measures of anatomical connectivity, rs-fMRI data have often been used to measure interregional functional connectivity. Typically, low-frequency (<0.1 Hz) oscillations in blood oxygenation level-dependent (BOLD) contrast are measured at each cortical and subcortical region over the course of 10–20 min, while the subject lies quietly in the scanner. Functional connectivity is estimated simply by the correlation (or partial correlation, or mutual information, or coherence) between each pair of regional BOLD time series, to constitute a matrix or functional connectome representing the strength of symmetrically coupled oscillations between all possible pairs of regions. It has been repeatedly demonstrated, by independent component analysis and other multivariate methods, that the human fMRI connectome has a characteristic anatomical pattern, with positively connected areas clustering in laterally symmetrical, functionally specialized resting-state networks, e.g., the frontoparietal network, comprising dlPFC, which may be positively or negatively connected to other resting-state networks, e.g., the DMN, comprising medial PFC and posterior cingulate cortex [20, 128] (see Fig. 5). It seems likely, given the reliability of functional connectomes measured repeatedly in the same person over time, and the replicability of functional connectome organization measured in different people and samples, that functional connectivity reflects some enduring substrate of monosynaptic connectivity [143]. However, the mechanisms by which anatomical connectivity might constrain functional connectivity are not yet certainly established. Much depends empirically on how anatomical connectivity is measured, e.g., by dMRI tractography, and how the rs-fMRI data are preprocessed in an effort to minimize noise and eliminate possible confounding effects of head motion prior to estimation of functional connectivity [144]. Experimental transection of interhemispheric callosal fibers in the macaque caused some disruption of functional connectivity between frontal regions, but this could be largely compensated by indirect anatomical connections mediated by intact anterior commissural fibers, implying that “a near-normal pattern of functional connectivity can be maintained by just a few indirect structural connections” [145]. However, careful studies using a seed-based correlational approach, whereby a single prefrontal cortical area is selected as a seed region and then functional connectivity is estimated between it and some or all other regions in the brain, have demonstrated that frontal areas defined by dMRI tractography in the human brain have distinct functional connectivity profiles that are somewhat consistent with the fMRI connectivity of homologous PFC regions in NHP [146, 147] (Fig. 5).

Taken together, there is clearly no single perfect MRI surrogate marker of monosynaptic connectivity, as it is measured directly by tract tracing. Indeed, all of these summarized MRI methods share the limitation of measuring undirected associations between regions, in place of the directed tracts defined by anterograde or retrograde tracers. The anatomical interpretation of MRI-derived connectivity metrics as proxies for monosynaptic connectivity between regions seems intuitively most straightforward for dMRI tractography, but there are technical limits to the resolution of long-distance tracts or crossing fibers by tractography. Morphometric similarity can be interpreted as a marker of monosynaptic connectivity, by two experimentally testable assumptions, but this relatively recent technique needs further development and validation. Structural covariance is also arguably interpretable as a marker of monosynaptic connectivity, but this technique suffers from the severe limitation that it can only be used to estimate mean anatomical connectivity in a group of brains. Rs-fMRI reflects some aspects of anatomical connectivity, but the mechanistic relationships between monosynaptic connectivity and coupled neurovascular oscillations are not entirely understood. Nonetheless, rs-fMRI continues to be very widely used for estimating the functional connectome of humans of all ages and across a wide range of disorders and has played a central role in understanding brain networks beyond monosynaptic connectivity. It has provided an approach to identify large, distributed networks, changes in which are linked to circuit dysfunction associated with mental health illnesses. Moreover, coupled with computational methods, described below, it allows for the identification of nodes and connectional hubs that likely are “critical gateways” for distributing information across whole-brain networks, as originally suggested by Mesulam.

## Connectomics: topological properties of brain networks modeled as graphs

Graph theory is a way of thinking formally about the topology of networks and the patterns of connections or edges between nodes that are conserved under any continuous spatial deformation or scaling. Mathematically, modern graph theory draws on ideas about topological analysis dating back to Euler in the seventeenth century and foundational studies of random graphs by Erdős, Rényi, and others in the twentieth century. In this century, graph theoretical tools have been very extensively developed and applied to quantitative analysis of diverse real-world networks or systems, ranging from social and economic networks to gene expression and protein interaction networks [148, 149].

One reason graph theory has been so central to the dramatic growth of twenty-first century network science is that graphs can be very simple, reducing a complicated and detailed real-life system to a stripped-down scheme of nodes and edges. Their simplicity makes graphs easier to visualize and quantify than the real systems they represent, and it makes them computationally feasible even for systems incorporating millions of nodes (and therefore trillions of edges). The other principal reason for the success of graph theory is that, despite their sometimes extreme simplicity, graphs have proven to be highly informative about the complex topological patterns that are nearly ubiquitous among social networks, economies and markets, nervous, and neurovascular systems [150]. In particular, graphical analysis of diverse real-world systems has consistently identified the existence of *hubs*, nodes that are especially central in some sense to the topological integration of the network as a whole.

Here, we will focus on brain network analysis—also known as connectomics—through the prism of the graph theoretical concept of hubs in brain networks. We will provide a brief introduction to degree centrality and related graph metrics of “hubness” that have been measured in many scales, modalities, and species of brain network data [151]. We will illustrate how hubness is related to other topological properties of the connectome, e.g., modules, and to anatomical constraints, e.g., spatial distance. For more comprehensive reviews of graph theoretical methods for network neuroscience, see [11, 152].

### Degree centrality of binary graphs: the simplest hubs

The minimal representation of a connectome is as a binary graph: a set of identical nodes connected to each other by a set of undirected, unweighted edges. An obvious way of identifying a highly connected hub in a binary graph is simply to count the number of edges that connect each node to the rest of the network. This amounts to estimating the degree of each node, or its degree centrality. Each node can then be arbitrarily designated a hub if its degree is in the top 5% or 10%, say, of the distribution of degree over all nodes in the binary network.

The degree distribution of binary brain graphs is not Normal. Like many other complex systems, the degree distribution of brain networks is asymmetrically long-tailed, often conforming technically to a truncated power law, so that the probability of a few nodes having an outstandingly high degree is much greater than would be expected in a random Normal graph [151, 153]. There is clear evidence for the existence of hubs, defined by degree criteria, in binary graphs of nervous systems ranging from the *Caenorhabditis elegans* synaptic–neuronal connectome [154] to the human dMRI tractography connectome [155].

However, this simplest concept of hubness is by no means the only, or always the best, possible metric. There, is in fact, a wealth of topological centrality (hubness) metrics available, which may be more or less appropriate, depending on the nature of the data available (tract tracing or MRI) and the hypothetical question of interest.

### Weighted and directed degree centrality metrics of hubness

We can define the connectome as a (*p* × *p)* matrix, where *p* is the number of nodes and each element of the matrix represents the edge, or pairwise association, between a pair of nodes *i* and *j*. This matrix will generally be weighted, meaning there is a continuously variable weight or strength of association for each edge, or element of the matrix. An unweighted or binary matrix can be constructed from a weighted matrix by applying a threshold to each edge so that weights greater than the threshold are binarised to 1, and weights less than the threshold go to 0. A weighted matrix can include edges that have negative (<0) and positive (>0) weights, in which case it may also be called a signed matrix; or it may only include edges with positive weights, in which case it is unsigned.

Weighted or binary, signed or unsigned, the connectome matrix can also be symmetric or asymmetric. In a symmetric matrix, the edge between *i* and *j* is identically weighted with the edge between *j* and *i*; thus, there are only (*p*2 − *p*)/2 unique, undirected edges in the connectome. In an asymmetric matrix, the edge from *i* to *j* can be differently weighted to the edge from *j* to *i*; so, there are twice as many (*p*2 − *p*) unique edges overall. An asymmetric connectome matrix will be drawn as a directed graph, with arrowheads on the edges.

For each of these different types of the connectome, there are corresponding measures of degree centrality. For weighted connectomes, the weighted degree is most simply the sum of the edges connecting each node to all other nodes, i.e., the sum of each row (or column) of the interregional correlation matrix from an rs-fMRI experiment. For asymmetric connectomes, the total degree of each node can be decomposed into the total number of efferent edges, or out-degree, and the number of afferent edges, or in-degree. For signed connectomes, the degree is either estimated separately for positive and negative weights or the degree is estimated from the sum of the absolute weights, thus effectively transforming a signed matrix to a (simpler) unsigned matrix. For each connectome, there also many other metrics of centrality besides degree, e.g., eigenvector centrality, closeness centrality, etc. [11].

We know a priori that mammalian whole-brain anatomical networks would be best represented by a weighted, unsigned, asymmetric connectome, allowing for variably dense, invariably positive, but not necessarily reciprocal, axonally mediated, monosynaptic connectivity between regions. Tract-tracing data, using directionally specific anterograde or retrograde tracers, and high-resolution measurement of tracer signals, have been used to estimate asymmetric, weighted connectomes for the mouse [156], the rat [157], the marmoset, and the macaque monkey [158]. However, MRI data, as indicated above, do not allow clear differentiation of afferent from efferent connections to or from a given region, and they have low signal-to-noise data compared to tract-tracing data [159]. Thus, human brain anatomical connectomes measured from MRI data are forced to be unrealistically symmetric and to represent a narrower range of variability in edge weights, or connection densities, than the many orders of magnitude variation in asymmetric axonal connection density that can be measured from tract-tracing data [160].

### Hubs, modules, and network integration

A less formal way of thinking about hubs in brain networks is by analogy to the global airline network [11]. If each airport is regarded as a node, and each flight contributes to the weight of the directed edge between nodes, then the global airline network can be represented as a weighted, directed graph. This network can be decomposed into a number of modules, or subnetworks, roughly corresponding to distinct geographical regions or continents. In general, the module of a graph comprises a community of nodes that are densely connected to each other, but sparsely connected to nodes in other modules or communities in the network. Various algorithms can be used to nearly decompose a graph into a number of subgraphs, communities, or modules defined in this way [149]. The modular community structure of the airline network reflects the well-known fact that most flights are between airports in the same regional module, e.g., domestic flights between US airports, and there are relatively few intercontinental flights or intermodular edges, e.g., long-haul flights between US and European airports.

The total hubness of each airport in the network can therefore be broken down in terms of its intramodular degree (the number of flights to other airports in the same module) and its intermodular degree (the number of flights to airports in different modules). Airports with a high intramodular degree can be called provincial hubs, like Anchorage in Alaska, because they have many local flights, whereas airports with high intermodular degree can be called connector hubs, like JFK in New York, because they have many intercontinental flights [161].

Brain networks generally also have a modular community structure [162]. This means the whole-brain graph can be nearly decomposed into a number of subgraphs or modules [163, 164]. In the connectomes of many different species, it has been found that topologically defined modules typically comprise anatomically neighboring nodes, collectively serving a specialized cognitive process. In mammalian connectomes, e.g., cortical areas serving specialist sensory input analysis are anatomically colocated in the occipital cortex and topologically affiliated to the same visual modules [165].

Thus, the hubness of each node in the connectome can be conditioned on the community structure in the same way as each airport in the global airline network. We can measure hubness in terms of intramodular and intermodular degree, or by the participation coefficient, which is a measure of the ratio between intra- and intermodular degree at each node [166]. In the mammalian cortex, areas with a high intramodular degree, a.k.a., provincial hubs, tend to be functionally specialized areas with many connections to nearby areas, whereas areas with a high intermodular degree, a.k.a., connector hubs, tend to be functionally generalized areas of association cortex with more connections to distant areas [167]. This observation of connector hubs in the human connectome is not only topologically analogous to the airline network it is also consistent with prior anatomical theories about the critical role of transmodal areas (heteromodal, paralimbic, and limbic cortices) in deriving cognition from sensation [168]: “transmodal areas are not necessarily centers where convergent knowledge resides, but critical gateways (hubs, sluices, or nexuses) for accessing the relevant, distributed information” from upstream areas of the more specialized sensory cortex [7].

In the brain, as in the global airline network, there is a fundamental tension between topological segregation and integration. In that sense, they are both examples of the broad class of complex networks defined by the “small-world” characteristic of high segregation and high integration compared to the topology of a random graph [169, 170]. Brains and airline networks combine both: (i) locally clustered, intramodular, or “cliquey” connectivity between nodes within a segregated subset of the network; and (ii) globally distributed, intermodular or “inclusive” connectivity between otherwise-segregated subsets of nodes that are thus integrated with the whole network. Provincial hubs are important for segregated topology and local clustering of connectivity between nodes in the same module; connector hubs are important for integrated topology and global connectivity between modules [9, 171]. Unlike the airline network, however, an emerging aspect of brain functional network organization is the capacity for some nodes to switch affiliation between modules dynamically over the course of time, leading to the concepts of dynamic connector hubs [172] and versatile modular affiliation [173].

For “higher-order” human brain function, and many neuropsychiatric disorders [174], it seems likely that topologically integrative features of the connectome, such as connector hubs, are particularly important. Higher-order, executive or intelligent functions are thought to be served by large-scale cortical or cortico-subcortical networks, encompassing regional nodes that are widely distributed in space across the brain and topologically affiliated to many different modules [175]. For example, the emergence of a global workspace for higher-order, effortful processing theoretically “breaks modularity” of specialized, automatic processing [176, 177], and the functional coactivation of a multitask network distributed across multiple cortical lobes is critical for the general intelligence factor, *g*, or adaptive task control [98]. These prior theories are compatible with brain graph results showing that integrative hub regions in the mouse connectome (tract-tracing data [156]) were enriched for expression of genes known to be functionally important for learning, memory, and single organism behavior, and in the *C. elegans* connectome (electron microscopy data [154]) most of the neurons constituting a “rich club” of densely interconnected hubs were the so-called command interneurons of the locomotor circuit with a functional role in backward or forward motion. Thus, in general, hubs seem to be important for adaptive whole animal behavior. Likewise, in the human connectome, performance of effortful cognitive tasks was associated with more intermodular edges and high-degree connector hubs [178], and hubness of frontal cortical nodes in MRI (morphometric similarity) networks was positively correlated with verbal and non-verbal intelligence quotient in healthy young people [142]. Further analysis and modeling of the relationships between connectome topology and information processing capacity is an active focus of ongoing research [179]. For example, recent studies have used the connectome to specify the wiring diagram of links between processing nodes in a neuromorphic computing model of memory capacity, seeking to use the connectome to bridge the gap between computational neuroscience and artificial intelligence [180].

The importance of hubs for global brain network integrity is also evidenced by in silico studies of resilience to attack. If the nodes of a connectome are serially deleted at random then the global efficiency of the network—a measure of topological integration—will incrementally decrease with each deletion [181]. However, the rate of decrease in global efficiency is accelerated as a function of the number of nodes deleted if the network is subject to a targeted attack, focusing first on the highest degree hub in the network and then proceeding to delete nodes in decreasing order of hubness [153]. Analogously, the global efficiency of the airline network will be much more severely degraded by a targeted attack on connector hubs, like JFK in New York, than by random attack on less central airports. This observation that connectome hubs not only make the network globally integrated (a strength) they also make it vulnerable to attack (a weakness) is not only topologically analogous to the airline network but it is also consistent with prior anatomical theories about the critical role of transmodal hubs [168]: “paradoxically, they also provide “neural bottlenecks” in the sense that they constitute regions of maximum vulnerability for lesion-induced deficits in the pertinent cognitive domain” [7].

In clinical studies of brain disorders, it has been shown that pathological changes in gray matter are more likely to be concentrated in high-degree hubs of the normative connectome than in low-degree non-hubs [10, 182, 183]. The anatomical locations of the hubs impacted by different disorders seem likely to be somewhat disease specific. For example, schizophrenia was associated with reduced hubness of prefrontal cortical nodes, whereas Alzheimer’s disease was associated with reduced hubness of temporal cortical nodes [10, 183]. The common association between neurological and psychiatric disorders and MRI evidence of abnormality in brain network hubs could reflect the disruptive impact of the pathological attack on the integrative aspects of network topology that are important for higher-order function. However, it is also possible that hubs are especially vulnerable to pathological processes [184], as well as being more likely to generate symptoms once lesioned [12, 175]. For example, in Alzheimer’s disease and other neurodegenerative disorders, the anatomical distribution of loci of gray matter atrophy (MRI “lesions”) has been mapped to the normative connectome and modeled as the outcome of various candidate pathogenic processes, including trans-synaptic propagation of a pathogenic agent from an initial epicenter, typically a network hub [185, 186]. Using a directed human anatomical connectome, obtained by combining tract-tracing data on the mouse brain with diffusion-weighted MRI data on selected cortico-subcortical connections of the human brain [187], it was recently reported that anterograde diffusion of a pathogenic agent provided a good account of the observed longitudinal progression of gray matter lesions in Huntington’s disease [188]. These and related recent studies [189] reinforce the importance of network hubs in shaping the anatomical progression of neurodegenerative processes and provide an elegant illustration of how descriptive connectomics can be used as the basis for more causal mechanistic models of brain disease. In this light, neurodegenerative processes can even be regarded as “natural experiments” in human tract tracing, whereby close correspondence between observed anatomical patterns of neurodegeneration and the patterns predicted by trans-synaptic propagation across the connectome supports the fundamental concept that hubs identified by statistical analysis of MRI data correspond to human brain regions with a high degree of axo-synaptic connectivity.

### Spatial embedding, wiring cost, and hubs

One reason that the connectome and the global airline network share so many topological features in common is that both are examples of complex networks embedded in space and spatial networks generally are constrained to reduce the costs associated with long-distance connections [190, 191]. The importance of minimizing the wiring cost of connectivity between neurons was first recognized by Ramón y Cajal, who proposed a small number of conservation laws, including conservation of space and conservation of biological material, which accounted for many details of neuronal histology and network configuration [192]. It has since been widely agreed that many aspects of brain network organization are consistent with minimization of wiring [193] and this parsimonious tendency is explicable in terms of the imperative to control metabolic costs and to fit a topologically high dimensional brain network into the finite and low-dimensional space of the skull [191, 194].

However, cost minimization cannot account entirely for brain network organization. The frequency distributions of connection distance in the *C. elegans*, mouse, macaque, and human connectomes generally indicate a majority of short-distance connections, but also more long-distance connections than would be expected in comparable networks generated by strict cost minimization rules [156, 195]. Moreover, long-distance connections tend to be concentrated on high-degree hubs, especially connector hubs mediating intermodular connections between spatially distributed modules [166]. These observations have motivated the concept that brain networks represent an economical trade-off between cost minimization (which drives the formation of anatomically colocated clusters and modules of densely interconnected nodes) and topological integration (which is valuable for information processing, but requires the existence of connector hubs and long-distance edges) [181, 196]. For example, connector hubs of the PFC will have more long-distance connections, e.g., to the parietal, temporal and posterior cingulate cortex, as described above.

Networks that accurately simulate the topological properties of connectomes can be produced by generative models that define the probability of a connection between two nodes as the product of a distance-penalizing, cost minimization factor, and a competitive factor that promotes the formation of connections between topologically similar nodes (homophily) regardless of the distance between them [197, 198]. It is possible that other selection pressures, such as the importance of resilience of brain networks to pathological attack, may also be important in the evolution of connectome topology. However, these results from generative modeling of normative network formation by a simple two-factor model (representing the trade-off between wiring cost and topological homophily) have been successfully extended to consideration of atypical connectomes associated with schizophrenia or other neurodevelopmental disorders, indicating the potential in future to understand more deeply the developmental mechanisms that determine the formation of clinically symptomatic or cognitively disabling human brain networks [199].

## Relationship between anatomic studies demonstrating hard wiring and MRI studies demonstrating networks and hubs

The relationship between structure and function is of ongoing interest. There is little doubt that diffusion tractography should reflect, at least partially, the anatomic hard wiring of brain regions. In contrast, while rs-fMRI is affected by structural constraints, it reflects the dynamic interactions of a network over time, and, as such, is expected to demonstrated connectivity beyond those constraints [200]. Structure/function comparisons are often made directly between dMRI and rs-fMRI, each of which has intrinsic problems in reflecting the actual hard wiring (as described above), or between the anatomic literature and MRI, which we will focus on first.

Early studies benchmarked the accuracy of human dMRI-derived maps by comparing tractography streamlines with the human and NHP anatomic literature [201–205]. As methods for high-quality scanning have improved, comparisons are increasingly being carried out between NHP dMRI-derived networks and the NHP tract-tracing literature [206–210]. However, relying on the literature, while accurate from an anatomic perspective and thus an important first step, is nonetheless limited. Most animal tracing studies focus primarily on the source and target endpoints (injection site and labeled cell distributions), with little precise information about the trajectories of the axons that connect the endpoints. In addition, most studies do not provide a comprehensive account of all connections to or from a specific area, limiting the documentation to selected areas of interest. However, when a comprehensive tracing experimental data set is compared to dMRI, the results are moderately consistent across modalities [158]. However, the PFC has not been extensively explored in this manner. More recently, studies have been designed specifically to compare anatomic pathways with dMRI-generated streamlines in NHPs [24, 68, 133, 211–214]. These data, most of which have been collected in independent groups of animals, have contributed to a better overall understanding of where and how dMRI streamlines can produce both false positives and false negatives.

Unlike dMRI, rs-fMRI is a measure of functional connectivity rather than structure. Thus, emphasis on understanding the relationship between tract-tracing data and rs-fMRI has not been viewed as critical. Nonetheless, several recent studies have begun to explore this relationship [215, 216]. Cross-species comparative rs-fMRI studies using data-driven approaches have now demonstrated that the distributed networks identified in the human brain could also be observed in NHPs [32, 84, 217–221]. Moreover, comparisons between published results from NHP tract-tracing studies and NHP rs-fMRI studies have demonstrated a link between hard wiring and functional connectivity [219, 220]. Taken together, functional connectivity patterns seen with rs-fMRI appear to be largely, but not completely, determined by the underlying anatomic connections [222, 223]. Interestingly, functional connections are most stable between regions with reciprocal structural connections [223]. In addition, a few studies have taken a region-based approach [215, 224–226], which also showed good correspondence between tract-tracing data and rs-fMRI connectivity and were more precise than comparisons between structural and functional MRI data [200, 227].

A relatively new approach to compare human rs-fMRI connectivity and NHP tract-tracer studies is to place seeds in human brain regions homologous to the injection sites for NHP experiments. In a study using rs-fMRI in humans, to examine whether there was a striatal hub at which projections from the inferior parietal lobule and PFC converged, seeds were placed in the caudate nucleus in homologous regions to specific tracer injections. Connectivity profiles of the seed-based circuits were consistent with the anatomical pattern of tracer connections to the homologous injection sites [226]. This type of study, in which specific injection sites are used for a seed-based correlational analysis, has great potential for identifying unique subregions within large anatomic areas that display hub-like characteristics.

However, even when comparing tract-tracing and MRI connectivity metrics in NHPs, two confounding elements remain: (i) the accuracy of registering the histological specimen with the MRI data to place the seed in precisely the same position as the tracer injection site; and (ii) individual variability across animals. Tract-tracing and MRI studies in the same animal allow for a voxel-wise assessment of tractography accuracy. Using a cross-species, cross-modal approach (NHP tracing and NHP dMRI in the same animal, combined with dMRI in humans), we can determine which streamlines were the correct ones and which represent false positives or false negatives. Below, we describe three studies that included some experiments in which tracer injections and the dMRI scan were carried out in the same animal. Two demonstrate the ability to understand the organization and trajectory of long-distance bundles carrying PFC fibers. The first describes the complex trajectory of the dopamine–PFC pathway; the second segments the ALIC based on the location of specific PFC fibers within it. The third study identifies and locates a hub within the larger rACC hub of the DMN [24, 28, 228].

### Ascending midbrain pathways

As described above, the MFB, which travels through the ventral forebrain, carries the ventral tegmental dopamine fibers to the PFC. Recently, a so-called new component of the MFB (referred to as the superolateral MFB) was described in the human brain based on dMRI tractography. This component appeared to travel within the IC, not in the classic MFB. Combining tracing and dMRI in the NHP, we could demonstrate that the streamlines entering the IC were false positives (Fig. 7). To reach the cortex, VTA fibers travel through the ventral forebrain, which could be demonstrated using dMRI and validated with tyrosine hydroxylase (TH)-positive immunoreactivity in both NHP and human brains. Other axons cross the IC to terminate in the caudal striatum (Fig. 7a). However, streamlines that enter the IC are false positives, which could also be demonstrated in the human brain (Fig. 7b, c). The lack of TH-positive fibers in the capsule further validates the lack of VTA dopamine fibers (and the MFB) passing through the capsule [28]. Thus, the long-distance dopaminergic pathway to the PFC does not travel through the IC, but rather follows the well-described MFB.

**Fig. 6:**
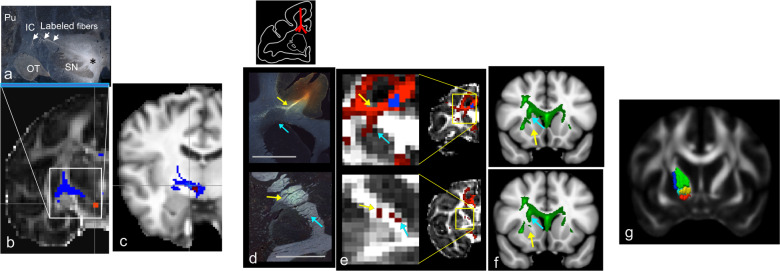
Tractography through the anterior limb of the medial forebrain bundle and ALIC. **a**–**c** Pathways through the MFB. **a** Asterisk indicates tracer injection site; **b**, **c** red dot indicates seed placement at the same site as the injection in monkey and human dMRI. Similar to the anatomic tracing, streamlines cross the internal capsule (IC) to the striatum. However, unlike the anatomic tracing experiment, streamlines also enter the IC and continue to travel rostrally, through the IC in both the monkey and human. **d**–**g** Pathways through the ALIC. **a** Histology showing fiber pathways following an injection site in the dorsal PFC. **b** NHP dMRI streamlines generated from a seed at the injection site location. Correct streamlines are indicated with yellow arrows, incorrect streamlines with blue arrows. **c** Human dMRI data illustrating streamlines following placement of a seed in a similar area of the dorsal PFC. Based on the NHP data, yellow arrows show the likely correct streamlines and blue arrows show the likely incorrect ones. **d** Organization of cortical fibers in the human ALIC. Red = vmPFC, yellow = ACC, teal = vlPFC, green = dmPFC, blue = dlPFC.

### PFC pathways through the ALIC

**Fig. 7:**
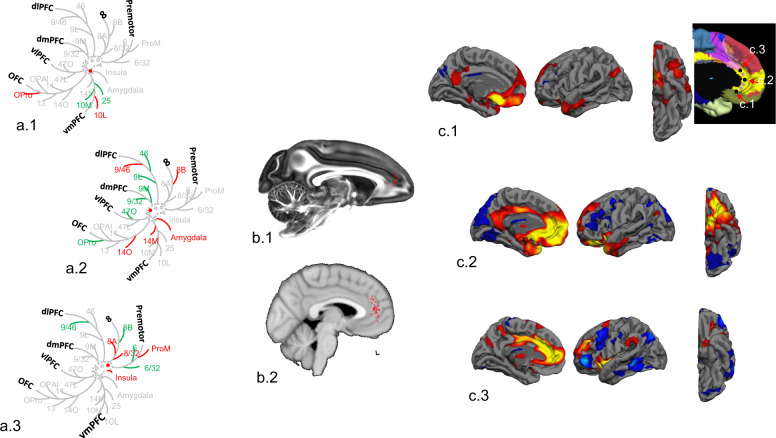
The rACC hub. **a** Schematic illustration of the FC regions with strong projections in each case. The dashed contour at the center of each schematic represents the rACC and the circles indicate the injection placement for each case. a.1 = injection 1, a.2 = injection 4, a.3 = injection 6. Colored branches represent the strength of inputs (based on cell counts) from each brain region (green = 50%; red = 75%). Case 4 (a.2) showed the most diverse input. **b** Top: sagittal section showing the localized hub in seven individual monkeys using dMRI tractography. Each red dot marks the center of the hub region in one monkey. The center of the hub was defined by the voxel with the highest weighted sum of probabilistic streamlines from all 29 seeded areas. Bottom: sagittal sections showing the localized hub across human individuals using dMRI tractography. Each red dot marks the center of the hub region in one subject. The center of the hub was defined by the voxel with the highest weighted − sum of probabilistic streamlines from all seeded FC areas. **c** rs-fMRI connectivity following seed placement in the rACC in similar locations as the anatomic injection sites. Top: seed placements. c.1. Seed placement in the ventral rACC. This placement showed the most limited connections, consistent with the NHP anatomy data. c.2. Seed placement in the central rACC. This placement showed the most diverse connections, consistent with the NHP anatomy data c.3. Seed placement in the dorsal rACC. This placement showed an intermediate level of connections, consistent with the NHP anatomy data.

As described above, the ALIC is organized such that fibers from dorsal PFC regions are dorsal to those from ventral regions, and axons from medial PFC regions are medial to those from lateral areas, which results in five segments: a ventral segment (vPFC fibers); medial and lateral midsegments (ACC and vlPFC fibers, respectively); and medial and lateral dorsal segments (medial PFC and dlPFC fibers) (see Fig. 3b) [55, 120]. Using the cross-species, cross-modal approach, it was possible to follow the correct streamlines and identify the false-positive streamlines as they split from the stalk and curved around the striatum to enter the ALIC (Fig. 7d, e). Not surprisingly, the same false positives were also found in the human dMRI (Fig. 7f). Moreover, these false positives were in the same positions as those seen in the NHP, demonstrating that using tractography with seeds placed precisely in the same position as the tracer injections shows precisely where the streamlines diverge from the actual pathway [24]. With this information, it was possible to follow the correct streamlines and show that the organization and topology of the ALIC are similar in the human ALIC (Fig. 7g) [68, 120]. This topological invariance is the critical feature when translating from animal studies to the human brain, which has much greater individual variability. Thus, although the precise location of the bundles in standard anatomical space is unlikely to be consistent across subjects, the relative position of the bundles within a subject is conserved. Understanding this organization sets the stage for linking abnormalities within the ALIC to specific connections and helps to fine-tune the neurosurgical placement of DBS electrodes [24, 28, 55, 120]. This type of multimodal, multispecies comparative study can be generalized to determine the organization of long-distance connections related to specific networks.

### An rACC hub

As indicated above, the rACC sits at the connectional intersection of the emotion, cognition, and executive control networks and is considered a global network hub and a key anchor in the DMN [83, 229]. However, this large region receives a gradient of inputs across the structure, such that the ventral part is linked to emotion processing areas, the rostral region with cognitive control areas, and the caudal regions with motor control areas [77, 230]. Thus, a key question is whether a hub exists within this gradient or whether the information is processed sequentially—i.e., from valuation to cognition to action. Based on the anatomic distribution of afferent connections to the rACC, we recently found that one subregion, located in the rACC and dACC, received inputs from more areas than would be expected based solely on the inputs predicted by anatomical gradients (dlPFC, vlPFC). The additional inputs include those from the OFC, FEFs, and vmPFC. These results suggest a hub embedded within the large rACC region of the DMN (Fig. 7a) [82]. In contrast, the most ventral rACC is most closely linked to the OFC and vmPFC, and thus the limbic network. Interestingly, the most dorsal and caudal area is linked to the FEFs and the insula, suggesting that this is part of the SN.

We tested whether we could identify this anatomically localized hub region using dMRI, first in NHPs and then in the human brain (Fig. 7b). Using each frontal voxel as a seed, tractography in the NHPs demonstrated that streamlines from each frontal area converged in a similar location within the rACC as the hub defined by tract-tracing experiments. Applying the same tractography approach to human connectome dMRI data from the Human Connectome Project, streamlines from the homologous frontal areas converged in a similar position in the human rACC. Interestingly, the variance across individuals was similar in both species, with little rostrocaudal variance, but greater dorsoventral variance.

Finally, we tested how well the anatomic results compared to rs-fMRI connectivity. Seeds were placed in the rACC at similar locations as anatomic injection sites in the NHP anatomy experiments. Consistent with the anatomic results, the most ventral seed resulted in relatively limited connections, while the central seed showed the most diverse connections. Connections of the ventral seed were strongest with the ventral medial parts of the PFC (areas 25, 10, and 14). Its main long-distance connection was to the PCC, consistent with its affiliation as part of the DMN. The anatomical pathway of this connection is through the CB [231]. The most dorsal seed is also tightly linked to adjacent areas (dlPFC and dmPFC) with long-distance connections to the premotor cortex (via the CB) and the frontoinsular cortex (FIC), specifically the insula. The anatomical pathway between the FIC and ACC is through the Extm [47]. Interestingly, this seed did not show strong connections to the PCC, one of the hubs of the DMN, suggesting that this position of the rACC may not be central to the DMN. However, its connections were more consistent with involvement in the SN [16] (see Menon and D’Esposito in this volume). The connections of the central seed, placed in a similar position as the rACC hub described above, had a wider range of long-distance connections, compared to the more dorsal and ventral seeds, including to the OFC, FEFs, vlPFC, and rostral temporal cortex, in addition to its strong connection to the PCC. The anatomical pathways mediating these diverse connections include: the Extm, to temporal cortex; the CB, to FEFs; and CB and UF to OFC [47, 231]. Taken together, the combination of anatomy and imaging allows us more specifically to pinpoint the location and composition of hubs and edges embedded within the network of larger regions initially identified using rs-fMRI.

Linking anatomy with imaging is a two-way street. While anatomy provides the ground truth for hard-wired connections, imaging can guide the interpretation of hard wiring. For example, in the above example of the rACC, the idea that this region is an important hub was identified by rs-fMRI [83], but then further probed by anatomic studies [82]. Combining the NHP anatomy and MRI data also lends validity to the location and composition of hubs and edges in the human connectome and eliminates issues related to cross-species, cross-modalities evaluation in one step [232]. These three studies show the value of NHP tract-tracing data for anatomically validating the connections defined by MRI that are likely to represent monosynaptic connections. However, this raises the issue of how to validate MRI-based connectivity without NHP tract-tracing data. Although the direct comparisons described above are ideal, they are available for all connections. However, there does exist extensive anatomic literature that can guide connectional studies.

Another way in which anatomical studies can be used to cross-validate the topological concept of hubness is by studying the physiology associated with cognitive or behavioral tasks at hubs or the consequences of focal lesions to hubs. For example, cells in the rACC hub region described above are sensitive to the value and uncertainty of aversive outcomes and actively anticipate the resolution of uncertainty about future risky outcomes [69] (see Monosov and Rushworth in this volume). As predicted by in silico models of network disintegration by a targeted attack on highly connected nodes, lesions of putative connector hubs of the cortex should have the greatest impact on complex behaviors that depend on the integration of information and action across multiple sensory and motor domains. For example, selective lesions of the sulcus of the ACC in NHPs caused behavioral deficits in reward learning, indicating an integrative role for the lesioned area in accessing reward probability information, widely distributed throughout the brain, as the cortical component of a distributed circuit to determine “which actions are worth making” [233]. Selective lesions of the gyrus of the ACC caused impairments in social behavior, consistent with it playing a crucial role in valuing social information on the basis of integrated interactions with several other brain areas including the OFC and amygdala [234]. These and other experimental lesion studies [235] suggest that locally targeted disruption of the cortex can have effects on extensively distributed information processing and related complex behaviors, as would be expected if the lesioned areas were topological hubs.

Most generally, these examples highlight the opportunity to use anatomic data—from tract-tracing and lesion studies—to demonstrate the hard wiring that underlies the concept of hubs based on human imaging. This paves the way to validate, probe, and explore in greater detail the relationship of anatomically and MRI-defined hubs to both normal and abnormal behaviors in future studies [235]. In this endeavor, a highly translational strategy, capable of working between microscale (cellular) and macroscale (whole brain), as well as between animal models and human studies, will be crucial to achieve a neuroscientifically panoramic view of hubs and other complex topological motifs in the frontal cortex.

## Conclusions

There is growing consensus that psychiatric illnesses are manifestations of circuit dysfunction. While, historically, anatomy has provided the foundation for understanding circuits, neuroimaging approaches have recently taken the lead. These approaches have demonstrated an intricate interplay in the complex network topology of the brain between segregation and integration, e.g., between functionally specialized modules and the connector hubs that bridge different modules. Highly connected hub nodes are central to network integration and MRI studies have demonstrated that they are also central to understanding many neurodegenerative and neurodevelopmental disorders. However, neuroimaging relies on indirect methods that often do not reflect the hard wiring that defines the underlying structure of networks. Now is a critical time for the field to circle back and evaluate how well the indirect methods of imaging reflect the hard wiring demonstrated by NHP tracing experiments. This knowledge forms the basis for identifying how circuits are organized and interact, and the extent to which connections observed in imaging studies represent direct wiring or second-order interactions and has important implications for understanding network interactions and therapeutic interventions. This review discussed NHP anatomy, imaging approaches and analyses, and imaging studies in both NHPs and humans that link across species and modalities. Taken together, these studies begin to address how closely structural (or functional) connectivity derived from MRI corresponds to the “gold standard” monosynaptic connectivity based on NHP tracing data. Further alignment between these two neuroscientific domains will be fundamental to establishing the biological mechanisms that underpin MRI measurements of frontal cortical hubs and their relevance for both higher-order cognitive function and its clinical disorders.

## References

[1] Brodmann K. Vergleichende Lokalisationslehre der Grosshirnrinde in ihren Prinzipien dargestellt auf Grund des Zellenbaues. Leipzig: Barth; 1909. p. x, 324.

[2] Vogt O, Vogt C (1919). Ergebnisse unserer Hirnforschung. J Psychol Neurol.

[3] Nieuwenhuys R (2013). The myeloarchitectonic studies on the human cerebral cortex of the Vogt-Vogt school, and their significance for the interpretation of functional neuroimaging data. Brain Struct Funct.

[4] Zola-Morgan S (1995). Localization of brain function: the legacy of Franz Joseph Gall (1758-1828). Annu Rev Neurosci.

[5] Catani M, Ffytche DH (2005). The rises and falls of disconnection syndromes. Brain.

[6] Geschwind N (1965). Disconnexion syndromes in animals and man. Brain.

[7] Mesulam MM (1998). From sensation to cognition. Brain.

[8] Raichle ME (2015). The brain’s default mode network. Annu Rev Neurosci.

[9] Gordon EM, Lynch CJ, Gratton C, Laumann TO, Gilmore AW, Greene DJ (2018). Three distinct sets of connector hubs integrate human brain function. Cell Rep.

[10] Crossley NA, Mechelli A, Scott J, Carletti F, Fox PT, McGuire P (2014). The hubs of the human connectome are generally implicated in the anatomy of brain disorders. Brain.

[11] Fornito A, Zalesky A, Bullmore E. Fundamentals of brain network analysis. San Diego: Academic Press; 2016.

[12] Fornito A, Zalesky A, Breakspear M (2015). The connectomics of brain disorders. Nat Rev Neurosci.

[13] Fair DA, Cohen AL, Dosenbach NU, Church JA, Miezin FM, Barch DM (2008). The maturing architecture of the brain’s default network. Proc Natl Acad Sci USA.

[14] van den Heuvel OA, Remijnse PL, Mataix-Cols D, Vrenken H, Groenewegen HJ, Uylings HB (2009). The major symptom dimensions of obsessive-compulsive disorder are mediated by partially distinct neural systems. Brain.

[15] Zhang Y, Suo X, Ding H, Liang M, Yu C, Qin W (2019). Structural connectivity profile supports laterality of the salience network. Hum Brain Mapp.

[16] Menon V, Uddin LQ (2010). Saliency, switching, attention and control: a network model of insula function. Brain Struct Funct.

[17] Uddin LQ (2015). Salience processing and insular cortical function and dysfunction. Nat Rev Neurosci.

[18] Corbetta M, Shulman GL (2011). Spatial neglect and attention networks. Annu Rev Neurosci.

[19] Farrant K, Uddin LQ (2015). Asymmetric development of dorsal and ventral attention networks in the human brain. Dev Cogn Neurosci.

[20] Yeo BT, Krienen FM, Sepulcre J, Sabuncu MR, Lashkari D, Hollinshead M (2011). The organization of the human cerebral cortex estimated by intrinsic functional connectivity. J Neurophysiol.

[21] Andrews-Hanna JR, Reidler JS, Sepulcre J, Poulin R, Buckner RL (2010). Functional-anatomic fractionation of the brain’s default network. Neuron.

[22] Li N, Baldermann JC, Kibleur A, Treu S, Akram H, Elias GJB (2020). A unified connectomic target for deep brain stimulation in obsessive-compulsive disorder. Nat Commun.

[23] Greenberg BD, Gabriels LA, Malone DA, Rezai AR, Friehs GM, Okun MS (2010). Deep brain stimulation of the ventral internal capsule/ventral striatum for obsessive-compulsive disorder: worldwide experience. Mol Psychiatry.

[24] Safadi Z, Grisot G, Jbabdi S, Behrens TE, Heilbronner SR, McLaughlin NCR (2018). Functional segmentation of the anterior limb of the internal capsule: linking white matter abnormalities to specific connections. J Neurosci.

[25] Pinhal CM, van den Boom BJG, Santana-Kragelund F, Fellinger L, Bech P, Hamelink R (2018). Differential effects of deep brain stimulation of the internal capsule and the striatum on excessive grooming in Sapap3 mutant mice. Biol Psychiatry.

[26] Liebrand LC, Caan MWA, Schuurman PR, van den Munckhof P, Figee M, Denys D (2019). Individual white matter bundle trajectories are associated with deep brain stimulation response in obsessive-compulsive disorder. Brain Stimul.

[27] Rasmussen SA, Noren G, Greenberg BD, Marsland R, McLaughlin NC, Malloy PJ (2018). Gamma ventral capsulotomy in intractable obsessive-compulsive disorder. Biol Psychiatry.

[28] Haber SN, Yendiki A, Jbabdi S. Four deep brain stimulation targets for obsessive-compulsive disorder: Are they different? Biol. Psych. 2020. 10.1016/j.biopsych.2020.06.031. 10.1016/j.biopsych.2020.06.031 PMC9569132 32951818

[29] Uddin LQ, Kelly AM, Biswal BB, Castellanos FX, Milham MP (2009). Functional connectivity of default mode network components: correlation, anticorrelation, and causality. Hum Brain Mapp.

[30] Margulies DS, Kelly AM, Uddin LQ, Biswal BB, Castellanos FX, Milham MP (2007). Mapping the functional connectivity of anterior cingulate cortex. Neuroimage.

[31] Koch SB, van Zuiden M, Nawijn L, Frijling JL, Veltman DJ, Olff M (2016). Aberrant resting-state brain activity in posttraumatic stress disorder: a meta-analysis and systematic review. Depress Anxiety.

[32] Liu C, Yen CC, Szczupak D, Ye FQ, Leopold DA, Silva AC (2019). Anatomical and functional investigation of the marmoset default mode network. Nat Commun.

[33] Whitfield-Gabrieli S, Ford JM (2012). Default mode network activity and connectivity in psychopathology. Annu Rev Clin Psychol.

[34] Philip NS, Carpenter SL, Sweet, LH Developing neuroimaging phenotypes of the default mode network in PTSD: integrating the resting state, working memory, and structural connectivity. J Vis Exp. 2014. 10.3791/51651. 10.3791/51651 PMC4210221 25046537

[35] Janowski V, Camerer C, Rangel A (2013). Empathic choice involves vmPFC value signals that are modulated by social processing implemented in IPL. Soc Cogn Affect Neurosci.

[36] Cooper JC, Kreps TA, Wiebe T, Pirkl T, Knutson B (2010). When giving is good: ventromedial prefrontal cortex activation for others’ intentions. Neuron.

[37] Milad MR, Quinn BT, Pitman RK, Orr SP, Fischl B, Rauch SL (2005). Thickness of ventromedial prefrontal cortex in humans is correlated with extinction memory. Proc Natl Acad Sci USA.

[38] Seo D, Lacadie CM, Tuit K, Hong KI, Constable RT, Sinha R (2013). Disrupted ventromedial prefrontal function, alcohol craving, and subsequent relapse risk. JAMA Psychiatry.

[39] Sescousse G, Caldu X, Segura B, Dreher JC (2013). Processing of primary and secondary rewards: a quantitative meta-analysis and review of human functional neuroimaging studies. Neurosci Biobehav Rev.

[40] Battaglia S, Garofalo S, di Pellegrino G, Starita F (2020). Revaluing the role of vmPFC in the acquisition of pavlovian threat conditioning in humans. J Neurosci.

[41] Myers-Schulz B, Koenigs M (2012). Functional anatomy of ventromedial prefrontal cortex: implications for mood and anxiety disorders. Mol Psychiatry.

[42] Knutson B, Fong GW, Bennett SM, Adams CM, Hommer D (2003). A region of mesial prefrontal cortex tracks monetarily rewarding outcomes: characterization with rapid event-related fMRI. Neuroimage.

[43] Haber SN, Behrens TE (2014). The neural network underlying incentive-based learning: implications for interpreting circuit disruptions in psychiatric disorders. Neuron.

[44] Schmahmann J, Pandya D. Fiber pathways of the brain. Oxford: Oxford University Press. 2006.

[45] Cavada C, Company T, Tejedor J, Cruz-Rizzolo RJ, Reinoso-Suarez F (2000). The anatomical connections of the macaque monkey orbitofrontal cortex. A review. Cereb Cortex.

[46] Barbas H. In: Chauvel P, Delgado-Escueta AV, editors. Advances in neurology. New York: Raven Press; 1992. p. 91–115.

[47] Petrides M, Pandya DN (2007). Efferent association pathways from the rostral prefrontal cortex in the macaque monkey. J Neurosci.

[48] Petrides M, Pandya DN (2006). Efferent association pathways originating in the caudal prefrontal cortex in the macaque monkey. J Comp Neurol.

[49] Petrides M, Pandya DN (2002). Comparative cytoarchitectonic analysis of the human and the macaque ventrolateral prefrontal cortex and corticocortical connection patterns in the monkey. Eur J Neurosci.

[50] Gerbella M, Belmalih A, Borra E, Rozzi S, Luppino G (2010). Cortical connections of the macaque caudal ventrolateral prefrontal areas 45A and 45B. Cereb Cortex.

[51] Carmichael ST, Price JL (1996). Connectional networks within the orbital and medial prefrontal cortex of Macaque monkeys. J Comp Neurol.

[52] Ongur D, Price JL (2000). The organization of networks within the orbital and medial prefrontal cortex of rats, monkeys and humans. Cereb Cortex.

[53] Preuss TM, Goldman-Rakic PS (1989). Connections of the ventral granular frontal cortex of macaques with perisylvian and somatosensory areas: anatomical evidence for somatic representation in primate frontal association cortex. J Comp Neurol.

[54] Krieg W. Architectonics of the human cerebral fiber systems. Evanston: Brain Books; 1973.

[55] Lehman JF, Greenberg BD, McIntyre CC, Rasmussen SA, Haber SN (2011). Rules ventral prefrontal cortical axons use to reach their targets: implications for diffusion tensor imaging tractography and deep brain stimulation for psychiatric illness. J Neurosci.

[56] Heilbronner SR, Haber SN. Anterior cingulate pathways through the cingulum bundle: Implications for neuroimaging and psychosurgery. New Orleans: Society For Neuroscience; 2012.

[57] Barbas H, Pandya DN (1984). Topography of commissural fibers of the prefrontal cortex in the rhesus monkey. Exp Brain Res.

[58] Makris N, Kennedy DN, McInerney S, Sorensen AG, Wang R, Caviness VS (2005). Segmentation of subcomponents within the superior longitudinal fascicle in humans: a quantitative, in vivo, DT-MRI study. Cereb Cortex.

[59] Rudebeck PH, Saunders RC, Lundgren DA, Murray EA (2017). Specialized representations of value in the orbital and ventrolateral prefrontal cortex: desirability versus availability of outcomes. Neuron.

[60] Schoenbaum G, Roesch MR, Stalnaker TA, Takahashi YK (2009). A new perspective on the role of the orbitofrontal cortex in adaptive behaviour. Nat Rev Neurosci.

[61] Camille N, Tsuchida A, Fellows LK (2011). Double dissociation of stimulus-value and action-value learning in humans with orbitofrontal or anterior cingulate cortex damage. J Neurosci.

[62] Noonan MP, Chau BKH, Rushworth MFS, Fellows LK (2017). Contrasting effects of medial and lateral orbitofrontal cortex lesions on credit assignment and decision-making in humans. J Neurosci.

[63] Morecraft RJ, Geula C, Mesulam M-M (1992). Cytoarchitecture and neural afferents of orbitofronal cortex in the brain of the monkey. J Comp Neurol.

[64] Barbas H (2007). Specialized elements of orbitofrontal cortex in primates. Ann NY Acad Sci.

[65] Carmichael ST, Price JL (1995). Sensory and premotor connections of the orbital and medial prefrontal cortex of macaque monkeys. J Comp Neurol.

[66] Carmichael ST, Price JL (1995). Limbic connections of the orbital and medial prefrontal cortex in macaque monkeys. J Comp Neurol.

[67] Sescousse G, Redoute J, Dreher JC (2010). The architecture of reward value coding in the human orbitofrontal cortex. J Neurosci.

[68] Jbabdi S, Lehman JF, Haber SN, Behrens TE (2013). Human and monkey ventral prefrontal fibers use the same organizational principles to reach their targets: tracing versus tractography. J Neurosci.

[69] Monosov IE, Haber SN, Leuthardt EC, Jezzini A (2020). Anterior cingulate cortex and the control of dynamic behavior in primates. Curr Biol.

[70] Botvinick M, Braver T (2015). Motivation and cognitive control: from behavior to neural mechanism. Annu Rev Psychol.

[71] Kolling N, Wittmann MK, Behrens TE, Boorman ED, Mars RB, Rushworth MF (2016). Value, search, persistence and model updating in anterior cingulate cortex. Nat Neurosci.

[72] Etkin A, Buchel C, Gross JJ (2015). The neural bases of emotion regulation. Nat Rev Neurosci.

[73] Shenhav A, Cohen JD, Botvinick MM (2016). Dorsal anterior cingulate cortex and the value of control. Nat Neurosci.

[74] Etkin A, Egner T, Kalisch R (2011). Emotional processing in anterior cingulate and medial prefrontal cortex. Trends Cogn Sci.

[75] Camille N, Griffiths CA, Vo K, Fellows LK, Kable JW (2011). Ventromedial frontal lobe damage disrupts value maximization in humans. J Neurosci.

[76] Jocham G, Hunt LT, Near J, Behrens TE (2012). A mechanism for value-guided choice based on the excitation-inhibition balance in prefrontal cortex. Nat Neurosci.

[77] Morecraft RJ, Stilwell-Morecraft KS, Cipolloni PB, Ge J, McNeal DW, Pandya DN (2012). Cytoarchitecture and cortical connections of the anterior cingulate and adjacent somatomotor fields in the rhesus monkey. Brain Res Bull.

[78] Jiang J, Beck J, Heller K, Egner T (2015). An insula-frontostriatal network mediates flexible cognitive control by adaptively predicting changing control demands. Nat Commun.

[79] Kolling N, Scholl J, Chekroud A, Trier HA, Rushworth MFS (2018). Prospection, perseverance, and insight in sequential behavior. Neuron.

[80] White JK, Bromberg-Martin ES, Heilbronner SR, Zhang K, Pai J, Haber SN (2019). A neural network for information seeking. Nat Commun.

[81] Holroyd CB, Coles MG (2002). The neural basis of human error processing: reinforcement learning, dopamine, and the error-related negativity. Psychol Rev.

[82] Tang W, Jbabdi S, Zhu Z, Cottaar M, Grisot G, Lehman JF, et al. A connectional hub in the rostral anterior cingulate cortex links areas of emotion and cognitive control. Elife. 2019;8. 10.7554/eLife.43761. 10.7554/eLife.43761 PMC6624020 31215864

[83] Buckner RL, Sepulcre J, Talukdar T, Krienen FM, Liu H, Hedden T (2009). Cortical hubs revealed by intrinsic functional connectivity: mapping, assessment of stability, and relation to Alzheimer’s disease. J Neurosci.

[84] Buckner RL, DiNicola LM (2019). The brain’s default network: updated anatomy, physiology and evolving insights. Nat Rev Neurosci.

[85] Picard N, Strick PL (1996). Motor areas of the medial wall: a review of their location and functional activation. Cereb Cortex.

[86] Caruana F, Gerbella M, Avanzini P, Gozzo F, Pelliccia V, Mai R, et al. Motor and emotional behaviours elicited by electrical stimulation of the human cingulate cortex. Brain. 2018. 10.1093/brain/awy219. 10.1093/brain/awy219 30107501

[87] Green AE, Fugelsang JA, Kraemer DJ, Shamosh NA, Dunbar KN (2006). Frontopolar cortex mediates abstract integration in analogy. Brain Res.

[88] Okuda J, Fujii T, Ohtake H, Tsukiura T, Yamadori A, Frith CD (2007). Differential involvement of regions of rostral prefrontal cortex (Brodmann area 10) in time- and event-based prospective memory. Int J Psychophysiol.

[89] Mansouri FA, Freedman DJ, Buckley MJ (2020). Emergence of abstract rules in the primate brain. Nat Rev Neurosci.

[90] Tsujimoto S, Genovesio A, Wise SP (2011). Frontal pole cortex: encoding ends at the end of the endbrain. Trends Cogn Sci.

[91] Burman KJ, Reser DH, Yu HH, Rosa MG (2011). Cortical input to the frontal pole of the marmoset monkey. Cereb Cortex.

[92] Baddeley A (1992). Working memory: the interface between memory and cognition. J Cogn Neurosci.

[93] Baddeley AD. Working memory. Oxford: Oxford University Press; 1986.

[94] Blumenfeld RS, Ranganath C (2006). Dorsolateral prefrontal cortex promotes long-term memory formation through its role in working memory organization. J Neurosci.

[95] Owen AM, Evans AC, Petrides M (1996). Evidence for a two-stage model of spatial working memory processing within the lateral frontal cortex: a positron emission tomography study. Cereb Cortex.

[96] Goldman-Rakic PS (1995). Cellular basis of working memory. Neuron.

[97] D’Esposito M, Postle BR, Rypma B (2000). Prefrontal cortical contributions to working memory: evidence from event-related fMRI studies. Exp Brain Res.

[98] Cole MW, Reynolds JR, Power JD, Repovs G, Anticevic A, Braver TS (2013). Multi-task connectivity reveals flexible hubs for adaptive task control. Nat Neurosci.

[99] Mitchell DJ, Bell AH, Buckley MJ, Mitchell AS, Sallet J, Duncan J (2016). A putative multiple-demand system in the macaque brain. J Neurosci.

[100] Petrides M, Pandya DN (1999). Dorsolateral prefrontal cortex: comparative cytoarchitectonic analysis in the human and the macaque brain and corticocortical connection patterns. Eur J Neurosci.

[101] Petrides M, Pandya DN (1984). Projections to the fronal cortex from the posterior parietal region in the rhesus monkey. J Comp Neurol.

[102] Cavada C, Goldman-Rakic PS (1989). Posterior parietal cortex in rhesus monkey: II. Evidence for segregated corticocortical networks linking sensory and limbic areas with the frontal lobe. J Comp Neurol.

[103] Barbas H, Pandya DN (1989). Architecture and intrinsic connections of the prefrontal cortex in the rhesus monkey. J Comp Neurol.

[104] Andersen RA, Asanuma C, Essick G, Siegel RM (1990). Corticocortical connections of anatomically and physiologically defined subdivisions within the inferior parietal lobule. J Comp Neurol.

[105] Cohen JR, Berkman ET, Lieberman MD. Intentional and incidental self-control in ventrolateral prefrontal cortex. In: Stuss DT, Knight RT, editors. Principles of frontal lobe function. Ch. 25. Oxford: Oxford University Press; 2013. p. 417–40.

[106] Badre D, Wagner A (2007). Left ventrolateral cortex and the cognitive control of memory. Neuropsychologia.

[107] Han S, O’Connor AR, Eslick AN, Dobbins IG (2012). The role of left ventrolateral prefrontal cortex during episodic decisions: semantic elaboration or resolution of episodic interference?. J Cogn Neurosci.

[108] Aron AR, Robbins TW, Poldrack RA (2014). Inhibition and the right inferior frontal cortex: one decade on. Trends Cogn Sci.

[109] Rygula R, Walker SC, Clarke HF, Robbins TW, Roberts AC (2010). Differential contributions of the primate ventrolateral prefrontal and orbitofrontal cortex to serial reversal learning. J Neurosci.

[110] Dajani DR, Uddin LQ (2015). Demystifying cognitive flexibility: Implications for clinical and developmental neuroscience. Trends Neurosci.

[111] Dippel G, Beste C (2015). A causal role of the right inferior frontal cortex in implementing strategies for multi-component behaviour. Nat Commun.

[112] Corbetta M, Shulman GL (2002). Control of goal-directed and stimulus-driven attention in the brain. Nat Rev Neurosci.

[113] Frey S, Mackey S, Petrides M (2014). Cortico-cortical connections of areas 44 and 45B in the macaque monkey. Brain Lang.

[114] Borra E, Gerbella M, Rozzi S, Luppino G (2011). Anatomical evidence for the involvement of the macaque ventrolateral prefrontal area 12r in controlling goal-directed actions. J Neurosci.

[115] Saleem KS, Miller B, Price JL (2014). Subdivisions and connectional networks of the lateral prefrontal cortex in the macaque monkey. J Comp Neurol.

[116] Barbas H (1988). Anatomic organization of basoventral and mediodorsal visual recipient prefrontal regions in the Rhesus monkey. J Comp Neurol.

[117] Hickok G (2012). Computational neuroanatomy of speech production. Nat Rev Neurosci.

[118] Levy BJ, Wagner AD (2011). Cognitive control and right ventrolateral prefrontal cortex: reflexive reorienting, motor inhibition, and action updating. Ann NY Acad Sci.

[119] Xu KZ, Anderson BA, Emeric EE, Sali AW, Stuphorn V, Yantis S (2017). Neural basis of cognitive control over movement inhibition: human fMRI and primate electrophysiology evidence. Neuron.

[120] Safadi Z, Grisot G, Jbabdi S, Behrens TE, Heilbronner SR, McLaughlin NCR (2018). Functional segmentation of the anterior limb of the internal capsule: linking white matter abnormalities to specific connections. J. Neurosci..

[121] Lewis DA, Campbell MJ, Foote SL, Morrison JH (1986). The monoaminergic innervation of primate neocortex. Hum Neurobiol.

[122] Lewis DA, Campbell MJ, Foote SL, Goldstein M, Morrison JH (1987). The distribution of tyrosine hydroxylase-immunoreactive fibers in primate neocortex is widespread but regionally specific. J Neurosci.

[123] Nieuwenhuys R, Geeraedts LM, Veening JG (1982). The medial forebrain bundle of the rat. I. General introduction. J Comp Neurol.

[124] Levitt P, Rakic P, Goldman-Rakic P (1984). Region-specific distribution of catecholamine afferents in primate cerebral cortex: a fluorescence histochemical analysis. J Comp Neurol.

[125] Oades RD, Halliday GM (1987). Ventral tegmental (A10) system: neurobiology. 1. Anatomy and connectivity. Brain Res.

[126] Coenen VA, Bewernick BH, Kayser S, Kilian H, Bostrom J, Greschus S (2019). Superolateral medial forebrain bundle deep brain stimulation in major depression: a gateway trial. Neuropsychopharmacology.

[127] Coenen VA, Panksepp J, Hurwitz TA, Urbach H, Madler B (2012). Human medial forebrain bundle (MFB) and anterior thalamic radiation (ATR): imaging of two major subcortical pathways and the dynamic balance of opposite affects in understanding depression. J Neuropsychiatry Clin Neurosci.

[128] Damoiseaux JS, Rombouts SA, Barkhof F, Scheltens P, Stam CJ, Smith SM (2006). Consistent resting-state networks across healthy subjects. Proc Natl Acad Sci USA.

[129] Maier-Hein KH, Neher PF, Houde JC, Cote MA, Garyfallidis E, Zhong J (2017). The challenge of mapping the human connectome based on diffusion tractography. Nat Commun.

[130] Jbabdi S, Sotiropoulos SN, Haber SN, Van Essen DC, Behrens TE (2015). Measuring macroscopic brain connections in vivo. Nat Neurosci.

[131] Jbabdi S, Behrens TE, Smith SM (2010). Crossing fibres in tract-based spatial statistics. Neuroimage.

[132] Reveley C, Seth AK, Pierpaoli C, Silva AC, Yu D, Saunders RC (2015). Superficial white matter fiber systems impede detection of long-range cortical connections in diffusion MR tractography. Proc Natl Acad Sci USA.

[133] Thomas C, Ye FQ, Irfanoglu MO, Modi P, Saleem KS, Leopold DA (2014). Anatomical accuracy of brain connections derived from diffusion MRI tractography is inherently limited. Proc Natl Acad Sci USA.

[134] Jbabdi S, Johansen-Berg H (2011). Tractography: where do we go from here?. Brain Connect.

[135] Zielinski BA, Gennatas ED, Zhou J, Seeley WW (2010). Network-level structural covariance in the developing brain. Proc Natl Acad Sci USA.

[136] Alexander-Bloch A, Raznahan A, Bullmore E, Giedd J (2013). The convergence of maturational change and structural covariance in human cortical networks. J Neurosci.

[137] Alexander-Bloch A, Giedd JN, Bullmore ET (2013). Imaging structural co-variance between human brain regions. Nat Rev Neurosci.

[138] Paquola C, Vos De Wael R, Wagstyl K, Bethlehem RAI, Hong SJ, Seidlitz J (2019). Microstructural and functional gradients are increasingly dissociated in transmodal cortices. PLoS Biol.

[139] Paquola C, Bethlehem RAI, Seidlitz J, Wagstyl K, Romero-Garcia R, Whitaker KJ (2019). Shifts in myeloarchitecture characterise adolescent development of cortical gradients. Elife.

[140] Seidlitz J, Váša F, Shinn M, Romero-Garcia R, Whitaker KJ, Vértes PE (2018). Morphometric similarity networks detect microscale cortical organization and predict inter-individual cognitive variation. Neuron.

[141] Hilgetag CC, Medalla M, Beul SF, Barbas H (2016). The primate connectome in context: principles of connections of the cortical visual system. Neuroimage.

[142] Seidlitz J, Sponheim C, Glen D, Ye FQ, Saleem KS, Leopold DA (2018). A population MRI brain template and analysis tools for the macaque. Neuroimage.

[143] van den Heuvel MP, Mandl RC, Kahn RS, Hulshoff Pol HE (2009). Functionally linked resting-state networks reflect the underlying structural connectivity architecture of the human brain. Hum Brain Mapp.

[144] Power JD, Plitt M, Gotts SJ, Kundu P, Voon V, Bandettini PA (2018). Ridding fMRI data of motion-related influences: Removal of signals with distinct spatial and physical bases in multiecho data. Proc Natl Acad Sci USA.

[145] O’Reilly JX, Croxson PL, Jbabdi S, Sallet J, Noonan MP, Mars RB (2013). Causal effect of disconnection lesions on interhemispheric functional connectivity in rhesus monkeys. Proc Natl Acad Sci USA.

[146] Sallet J, Mars RB, Noonan MP, Neubert FX, Jbabdi S, O’Reilly JX (2013). The organization of dorsal frontal cortex in humans and macaques. J Neurosci.

[147] Neubert FX, Mars RB, Sallet J, Rushworth MF (2015). Connectivity reveals relationship of brain areas for reward-guided learning and decision making in human and monkey frontal cortex. Proc Natl Acad Sci USA.

[148] Albert R, Barabasi AL (2002). Statistical mechanics of complex networks. Rev Mod Phys.

[149] Newman ME. Networks - an introduction. Oxford: Oxford University Press; 2010.

[150] Ji X, Ferreira T, Friedman B, Liu R, Liechty H, Bas E, et al. Brain microvasculature has a common topology with local differences in geometry that match metabolic load. Neuron. 2021;109. 10.1016/j.neuron.2021.02.006. 10.1016/j.neuron.2021.02.006 PMC8525211 33657412

[151] van den Heuvel MP, Sporns O (2013). Network hubs in the human brain. Trends Cogn Sci.

[152] Sporns O. Networks of the brain. Cambridge: MIT Press; 2010.

[153] Achard S, Salvador R, Whitcher B, Suckling J, Bullmore E (2006). A resilient, low-frequency, small-world human brain functional network with highly connected association cortical hubs. J Neurosci.

[154] Towlson EK, Vértes PE, Ahnert SE, Schafer WR, Bullmore ET (2013). The rich club of the *C. elegans* neuronal connectome. J Neurosci.

[155] Hagmann P, Cammoun L, Gigandet X, Meuli R, Honey CJ, Wedeen VJ (2008). Mapping the structural core of human cerebral cortex. PLoS Biol.

[156] Rubinov M, Ypma RJ, Watson C, Bullmore ET (2015). Wiring cost and topological participation of the mouse brain connectome. Proc Natl Acad Sci USA.

[157] Bota M, Sporns O, Swanson LW (2015). Architecture of the cerebral cortical association connectome underlying cognition. Proc Natl Acad Sci USA.

[158] Markov NT, Ercsey-Ravasz MM, Ribeiro Gomes AR, Lamy C, Magrou L, Vezoli J (2014). A weighted and directed interareal connectivity matrix for macaque cerebral cortex. Cereb Cortex.

[159] Fornito A, Zalesky A, Breakspear M (2013). Graph analysis of the human connectome: promise, progress, and pitfalls. Neuroimage.

[160] Ypma RJ, Bullmore ET (2016). Statistical analysis of tract-tracing experiments demonstrates a dense, complex cortical network in the mouse. PLoS Comput Biol.

[161] Sales-Pardo M, Guimera R, Moreira AA, Amaral LAN (2007). Extracting the hierarchical organization of complex systems. Proc Natl Acad Sci USA.

[162] Meunier D, Lambiotte R, Bullmore ET (2010). Modular and hierarchically modular organization of brain networks. Front Neurosci.

[163] Sporns O (2013). Making sense of brain network data. Nat Methods.

[164] Newman ME (2006). Modularity and community structure in networks. Proc Natl Acad Sci USA.

[165] van den Heuvel MP, Bullmore ET, Sporns O (2016). Comparative connectomics. Trends Cogn Sci.

[166] Vértes PE, Rittman T, Whitaker KJ, Romero-Garcia R, Váša F, Kitzbichler MG (2016). Gene transcription profiles associated with inter-modular hubs and connection distance in human functional magnetic resonance imaging networks. Philos Trans R Soc Ser B.

[167] Crossley NA, Mechelli A, Vertes PE, Winton-Brown TT, Patel AX, Ginestet CE (2013). Cognitive relevance of the community structure of the human brain functional coactivation network. Proc Natl Acad Sci USA.

[168] Mesulam M (1994). Neurocognitive networks and selectively distributed processing. Rev Neurol.

[169] Watts DJ, Strogatz SH (1998). Collective dynamics of ‘small-world’ networks. Nature.

[170] Bassett DS, Bullmore ET (2017). Small-world brain networks revisited. Neuroscientist.

[171] Sporns O (2013). Network attributes for segregation and integration in the human brain. Curr Opin Neurobiol.

[172] Zalesky A, Fornito A, Cocchi L, Gollo LL, Breakspear M (2014). Time-resolved resting-state brain networks. Proc Natl Acad Sci USA.

[173] Shinn M, Romero-Garcia R, Seidlitz J, Vasa F, Vertes PE, Bullmore E (2017). Versatility of nodal affiliation to communities. Sci Rep.

[174] Siugzdaite R, Bathelt J, Holmes J, Astle DE (2020). Transdiagnostic brain mapping in developmental disorders. Curr Biol.

[175] van den Heuvel MP, Sporns O (2019). A cross-disorder connectome landscape of brain dysconnectivity. Nat Rev Neurosci.

[176] Dehaene S, Kerszberg M, Changeux JP (1998). A neuronal model of a global workspace in effortful cognitive tasks. Proc Natl Acad Sci USA.

[177] Fodor JA. The modularity of mind: an essay on faculty psychology. Cambridge: MIT Press; 1983.

[178] Kitzbichler MG, Henson RN, Smith ML, Nathan PJ, Bullmore ET (2011). Cognitive effort drives workspace configuration of human brain functional networks. J Neurosci.

[179] Mišić B, Sporns O (2016). From regions to connections and networks: new bridges between brain and behavior. Curr Opin Neurobiol.

[180] Suarez LE, Richard BA, Lajoi G, Misic B (2021). Learning function from structure in neuromorphic networks. Nat Mach Intell..

[181] Achard S, Bullmore E (2007). Efficiency and cost of economical brain functional networks. PLoS Comput Biol.

[182] Achard S, Delon-Martin C, Vertes PE, Renard F, Schenck M, Schneider F (2012). Hubs of brain functional networks are radically reorganized in comatose patients. Proc Natl Acad Sci USA.

[183] Morgan SE, Seidlitz J, Whitaker KJ, Romero-Garcia R, Clifton NE, Scarpazza C (2019). Cortical patterning of abnormal morphometric similarity in psychosis is associated with brain expression of schizophrenia-related genes. Proc Natl Acad Sci USA.

[184] de Haan W, Mott K, van Straaten EC, Scheltens P, Stam CJ (2012). Activity dependent degeneration explains hub vulnerability in Alzheimer’s disease. PLoS Comput Biol.

[185] Zhou J, Gennatas ED, Kramer JH, Miller BL, Seeley WW (2012). Predicting regional neurodegeneration from the healthy brain functional connectome. Neuron.

[186] Seeley WW. Mapping neurodegenerative disease onset and progression. Cold Spring Harb Perspect Biol. 2017. 9. 10.1101/cshperspect.a023622. 10.1101/cshperspect.a023622 PMC5538416 28289062

[187] Pandya S, Mezias C, Raj A (2017). Predictive model of spread of progressive supranuclear palsy using directional network diffusion. Front Neurol.

[188] Raj A, Powell F (2021). Network model of pathology spread recapitulates neurodegeneration and selective vulnerability in Huntington’s disease. Neuroimage.

[189] Cauda F, Mancuso L, Nani A, Ficco L, Premi E, Manuello J (2020). Hubs of long-distance co-alteration characterize brain pathology. Hum Brain Mapp.

[190] Barthélemy M (2011). Spatial networks. Phys Rep.

[191] Bassett DS, Greenfield DL, Meyer-Lindenberg A, Weinberger DR, Moore SW, Bullmore ET (2010). Efficient physical embedding of topologically complex information processing networks in brains and computer circuits. PLoS Comput Biol.

[192] Ramon y Cajal S. Histology of the nervous system of man and vertebrates. Oxford: Oxford University Press; 1995.

[193] Chen BL, Hall DH, Chklovskii DB (2006). Wiring optimization can relate neuronal structure and function. Proc Natl Acad Sci USA.

[194] Betzel RF, Medaglia JD, Papadopoulos L, Baum GL, Gur R, Gur R (2017). The modular organization of human anatomical brain networks: accounting for the cost of wiring. Netw Neurosci.

[195] Kaiser M, Hilgetag CC (2006). Nonoptimal component placement, but short processing paths, due to long-distance projections in neural systems. PLoS Comput Biol.

[196] Bullmore E, Sporns O (2012). The economy of brain network organization. Nat Rev Neurosci.

[197] Vértes PE, Alexander-Bloch AF, Gogtay N, Giedd JN, Rapoport JL, Bullmore ET (2012). Simple models of human brain functional networks. Proc Natl Acad Sci USA.

[198] Betzel RF, Avena-Koenigsberger A, Goñi J, He Y, De Reus MA, Griffa A (2016). Generative models of the human connectome. NeuroImage.

[199] Akarca D, Vértes PE, Bullmore ET, Astle DE (2021). A generative network model of neurodevelopment. Nat Commun.

[200] Honey CJ, Sporns O, Cammoun L, Gigandet X, Thiran JP, Meuli R (2009). Predicting human resting-state functional connectivity from structural connectivity. Proc Natl Acad Sci USA.

[201] Behrens TE, Woolrich MW, Jenkinson M, Johansen-Berg H, Nunes RG, Clare S (2003). Characterization and propagation of uncertainty in diffusion-weighted MR imaging. Magn Reson Med.

[202] Lehéricy S, Ducros M, Van de Moortele PF, Francois C, Thivard L, Poupon C (2004). Diffusion tensor fiber tracking shows distinct corticostriatal circuits in humans. Ann Neurol.

[203] Hofer S, Merboldt KD, Tammer R, Frahm J (2008). Rhesus monkey and human share a similar topography of the corpus callosum as revealed by diffusion tensor MRI in vivo. Cereb Cortex.

[204] Frey S, Campbell JS, Pike GB, Petrides M (2008). Dissociating the human language pathways with high angular resolution diffusion fiber tractography. J Neurosci.

[205] Leh SE, Ptito A, Chakravarty MM, Strafella AP (2007). Fronto-striatal connections in the human brain: a probabilistic diffusion tractography study. Neurosci Lett.

[206] Calabrese E, Badea A, Coe CL, Lubach GR, Shi Y, Styner MA (2015). A diffusion tensor MRI atlas of the postmortem rhesus macaque brain. Neuroimage.

[207] Feng L, Jeon T, Yu Q, Ouyang M, Peng Q, Mishra V (2017). Population-averaged macaque brain atlas with high-resolution ex vivo DTI integrated into in vivo space. Brain Struct Funct.

[208] Van Essen DC, Jbabdi S, Sotiropoulos SN, Chen C, Dikranian K, Coalson T, et al. Diffusion MRI for in vivo neuroanatomy. In: Heidi Johansen-Berg, Timothy EJ Behrens, editor. Diffusion MRI. 2nd ed. Amsterdam: Academic Press, 2014. p. 337–58.

[209] Thiebaut de Schotten M, Dell’Acqua F, Valabregue R, Catani M (2012). Monkey to human comparative anatomy of the frontal lobe association tracts. Cortex.

[210] Mars RB, Jbabdi S, Sallet J, O’Reilly JX, Croxson PL, Olivier E (2011). Diffusion-weighted imaging tractography-based parcellation of the human parietal cortex and comparison with human and macaque resting-state functional connectivity. J Neurosci.

[211] Folloni D, Sallet J, Khrapitchev AA, Sibson N, Verhagen L, Mars RB. Dichotomous organization of amygdala/temporal-prefrontal bundles in both humans and monkeys. Elife. 2019;8. 10.7554/eLife.47175. 10.7554/eLife.47175 PMC6831033 31689177

[212] Donahue CJ, Sotiropoulos SN, Jbabdi S, Hernandez-Fernandez M, Behrens TE, Dyrby TB (2016). Using diffusion tractography to predict cortical connection strength and distance: a quantitative comparison with tracers in the monkey. J Neurosci.

[213] Azadbakht H, Parkes LM, Haroon HA, Augath M, Logothetis NK, de Crespigny A (2015). Validation of high-resolution tractography against in vivo tracing in the macaque visual cortex. Cereb Cortex.

[214] Reid AT, Lewis J, Bezgin G, Khundrakpam B, Eickhoff SB, McIntosh AR (2016). A cross-modal, cross-species comparison of connectivity measures in the primate brain. Neuroimage.

[215] Kelly C, Uddin LQ, Shehzad Z, Margulies DS, Castellanos FX, Milham MP (2010). Broca’s region: linking human brain functional connectivity data and non-human primate tracing anatomy studies. Eur J Neurosci.

[216] Lopez-Persem A, Roumazeilles L, Folloni D, Marche K, Fouragnan EF, Khalighinejad N (2020). Differential functional connectivity underlying asymmetric reward-related activity in human and non-human primates. Proc Natl Acad Sci USA.

[217] Kojima T, Onoe H, Hikosaka K, Tsutsui K, Tsukada H, Watanabe M (2009). Default mode of brain activity demonstrated by positron emission tomography imaging in awake monkeys: higher rest-related than working memory-related activity in medial cortical areas. J Neurosci.

[218] Neubert FX, Mars RB, Thomas AG, Sallet J, Rushworth MF (2014). Comparison of human ventral frontal cortex areas for cognitive control and language with areas in monkey frontal cortex. Neuron.

[219] Hori Y, Schaeffer DJ, Gilbert KM, Hayrynen LK, Clery JC, Gati JS (2020). Comparison of resting-state functional connectivity in marmosets with tracer-based cellular connectivity. Neuroimage.

[220] Miranda-Dominguez O, Mills BD, Grayson D, Woodall A, Grant KA, Kroenke CD (2014). Bridging the gap between the human and macaque connectome: a quantitative comparison of global interspecies structure-function relationships and network topology. J Neurosci.

[221] Oligschlager S, Xu T, Baczkowski BM, Falkiewicz M, Falchier A, Linn G (2019). Gradients of connectivity distance in the cerebral cortex of the macaque monkey. Brain Struct Funct.

[222] Shen K, Bezgin G, Hutchison RM, Gati JS, Menon RS, Everling S (2012). Information processing architecture of functionally defined clusters in the macaque cortex. J Neurosci.

[223] Shen K, Hutchison RM, Bezgin G, Everling S, McIntosh AR (2015). Network structure shapes spontaneous functional connectivity dynamics. J Neurosci.

[224] Oler JA, Tromp DPM, Fox AS, Kovner R, Davidson RJ, Alexander AL (2017). Connectivity between the central nucleus of the amygdala and the bed nucleus of the stria terminalis in the non-human primate: neuronal tract tracing and developmental neuroimaging studies. Brain Struct Funct.

[225] Choi EY, Yeo BT, Buckner RL (2012). The organization of the human striatum estimated by intrinsic functional connectivity. J Neurophysiol.

[226] Choi EY, Tanimura Y, Vage PR, Yates EH, Haber SN (2017). Convergence of prefrontal and parietal anatomical projections in a connectional hub in the striatum. Neuroimage.

[227] Honey CJ, Thivierge JP, Sporns O (2010). Can structure predict function in the human brain?. Neuroimage.

[228] Tang W, Yendiki A, Jbabdi S, Haber S (2018). Location of anterior cingulate and ventrolateral prefrontal cortical hubs: integration between emotional and cognitive functions. Biol Psychiatry.

[229] Hagmann P, Cammoun L, Gigandet X, Gerhard S, Ellen Grant P, Wedeen V (2010). MR connectomics: principles and challenges. J Neurosci Methods.

[230] Vogt BA. In: BA Vogt, editor. Cingulate neurobiology and disease. Ch. 1. Oxford: Oxford University Press; 2009. p. 3–30.

[231] Heilbronner SR, Haber SN (2014). Frontal cortical and subcortical projections provide a basis for segmenting the cingulum bundle: implications for neuroimaging and psychiatric disorders. J Neurosci.

[232] Mars RB, Jbabdi S, Rushworth MFS. A common space approach to comparative neuroscience. Annu Rev Neurosci. 2021. 10.1146/annurev-neuro-100220-025942. 10.1146/annurev-neuro-100220-025942 PMC7618914 33534614

[233] Kennerley SW, Walton ME, Behrens TE, Buckley MJ, Rushworth MF (2006). Optimal decision making and the anterior cingulate cortex. Nat Neurosci.

[234] Rudebeck PH, Buckley MJ, Walton ME, Rushworth MF (2006). A role for the macaque anterior cingulate gyrus in social valuation. Science.

[235] Mansouri FA, Buckley MJ, Mahboubi M, Tanaka K (2015). Behavioral consequences of selective damage to frontal pole and posterior cingulate cortices. Proc Natl Acad Sci USA.

[236] Yeh CH, Jones DK, Liang X, Descoteaux M, Connelly A (2021). Mapping structural connectivity using diffusion MRI: challenges and opportunities. J Magn Reson Imaging.

